# Deficit saline water irrigation under reduced tillage and residue mulch improves soil health in sorghum-wheat cropping system in semi-arid region

**DOI:** 10.1038/s41598-020-80364-4

**Published:** 2021-01-21

**Authors:** Pooja Gupta Soni, Nirmalendu Basak, Arvind Kumar Rai, Parul Sundha, Bhaskar Narjary, Parveen Kumar, Gajender Yadav, Satyendra Kumar, Rajender Kumar Yadav

**Affiliations:** 1 ICAR-Central Soil Salinity Research Institute, Karnal, Haryana 132 001 India; 2 IARI-Krishi Vigyan Kendra, Shikohpur, Gurugram, Gurugram, Haryana 122 004 India

**Keywords:** Biogeochemistry, Ecology, Environmental sciences

## Abstract

Judicious application of saline water except for critical growth stages, could be the only practical solution to meet the crop water demand in arid and semi-arid regions, due to limited access to freshwater, especially during dry winter months. A field experiment was conducted to study the effect of tillage [conventional (CT), reduced (RT), and zero (ZT)], rice straw mulch and deficit saline-water irrigation in wheat (100, 80 and 60% of wheat water requirement, CWR) followed by rainfed sorghum on soil properties and the yields of the cropping system. Yields of both the crops were comparable between RT and CT, but the wheat yield was reduced in ZT. The RT, mulching and deficit saline irrigation in wheat season (60% CWR) increased the sorghum fodder yield. Olsen’s P (8.7–20.6%) and NH_4_OAc-K (2.5–7.5%) increased in RT and ZT, respectively, over CT under both the crops. Deficit irrigation reduced soil salinity (EC_e_) by 0.73–1.19 dS m^−1^ after each crop cycle, while soil microbial biomass C (MBC) and N (MBN), dehydrogenase, urease and alkaline phosphatase reduced with an increase in EC_e_. The *α*-glucosidase, MBC, EC_e_, KMnO_4_oxidizable N, and urease were identified as major contributors in developing the soil health index. Deficit irrigation (60% CWR) and rice straw mulching under ZT and RT showed higher values of soil health index. Overall, deficit saline-water irrigation under reduced tillage and straw mulching had the greatest potential in maintaining soil health, saving fresh irrigation water without affecting the productivity of the sorghum-wheat system in the semi-arid regions of India. Results also demonstrated that salt affected areas of arid and semiarid countries can replicate the protocol for indexing and screening of soil health indicators to assess the sustainability of a cropping system. This integrated management based on the nature of the available resources also provided a practical approach to achieve the target of land degradation neutrality and land restoration.

The development of integrated soil and crop management practices is important for the sustainable crop production and restoring soil health in semi-arid regions of the world^1,2^. Increasing population pressure calls for multifunctional use of available land and water resources within its system boundary to avoid further degradation and promote restoration^3,4^. The sustainability of agriculture in these regions is always under threat because of inherent soil salinity, scarcity of fresh water and dependency on saline-water for irrigation^5,6^. Low rainfall, high temperature and evapotranspiration demand favour root zone salinization^7,8^. A further buildup of salinity is through saline groundwater irrigation to meet the high crop water demand^9^. Wheat is a semi-salt tolerant crop grown as a staple food crop in the western Indo-Gangetic plains of India. Under the limited freshwater availability, saline groundwater can only meet the irrigation demand in wheat. However, the existing practice of applying 35 cm of irrigation water each of 7 cm depth^9^ at five critical stages cause root zone salinization in wheat, with associated negative impacts on crop productivity and Sustainable Development Goals related to food security (SDGs 2) in long term^4,7,10^.

Sorghum is a moderately salt-tolerant C_4_ grass adapted in rainfed semi-arid and arid regions^11^. Its salinity tolerance threshold is 6.86 dS m^−1^ with a slope of 16 per cent per dS m^−1^ of irrigation water^12^. It is an important *kharif* season (July–Oct) crop grown over a wide range of soil types under rainfed conditions for forage purposes^13^. Rainfed sorghum (*Sorghum bicolor* L. Moench)—irrigated wheat (*Triticum aestivum* L.) cropping system provides an opportunity for intra/inter-season root zone salinity management by promoting the leaching of salt in the rainy season.

Deficit irrigation and mulching are strategies for water-saving and minimize salt loading by reducing water applied at each irrigation event. Deficit irrigation with available saline-water is practiced to prevent frequent drought and sustain crop yield^14,15^. Deficit saline irrigation up to 65% of full irrigation requirement with water salinity of 7.5 dS m^−1^ produced wheat grain yield equivalent to full irrigation^16^. Rice straw mulch and efficient water management had the potential for improving soil quality, reducing evaporation and increasing crop productivity^17^. These practices allow water to move more quickly into and through the soil profile, improving salt leaching and inhibition of salt build-up in surface soil^18^. Therefore, the combined practice of deficit irrigation and mulching appears promising because of reduced salt loading through irrigation water as well as reduced upward salt flux. The deficit saline-water irrigation and mulching strategies synergized with climate and soil type of geographical unit can be a nature-based solution working within the boundaries of the available natural resources^3^. Conservation tillage practices like zero- and reduced tillage under water-limited environments is an attractive strategy to enable early planting, and maintaining wheat yield equivalent to conventional tillage^19^. Zero tillage is also effective in improving soil fertility, restoring soil organic carbon, soil structure and leads to a reduction in soil evaporation and salinity^19^.

Soil health index (SHI) has been developed under different agro-climate and agroecosystems^17,20^, such information is missing in saline soils. We hypothesized that the chemical and biological attributes of soil health and crop yield will respond differently to short-term effects of different tillage, irrigation practices (rainfed sorghum, deficit saline irrigation for wheat) and straw mulching. Further, a soil health index (SHIs) which is an integrated expression of most sensitive attributes, if found, could monitor soil health after sorghum/wheat in a holistic way for making the most sustainable management choices. To fill the gaps, a systematic study was conducted to (1) evaluate the combined effect of tillage, deficit saline irrigation, and mulching on crop yield; (2) identify key chemical and biological soil health indicators; and (3) to develop an SHI to identify the best practices for the sorghum-wheat system in a saline soil and water dominant area of north-west semi-arid India.

## Results

### Soil pH and electrical conductivity

The soil of experimental plots was slightly alkaline (pH > 7.0); however, soil electrical conductivity (EC_e_) was higher after *rabi* (8.87 and 6.41 dS m^−1^) compared to *kharif* (5.48 and 3.59 dS m^−1^) (Tables 1, 2, 3). The deficit irrigated treatments recorded the lowest value of EC_e_ compared to 100CWR (*P* < 0.05) (Supplementary Table 1). Mulch reduced salt load up to 7.35 dS m^−1^ compared to no mulch 7.92 dS m^−1^ in the soil after harvest of wheat (*P* < 0.05) (Table 1).

**Table 1:** Influence of tillage, irrigation and mulch on soil pH_s_, EC_e_ (dS m^−1^), Walkley and Black oxidizable organic C (WBOC; g kg^−1^) and KMnO_4 _oxidizable N, Olsen’s P and NH_4_OAc extractable K (kg ha^−1^).

Treatments	After sorghum						After wheat					
	pH_s_	EC_e_	WBOC	N	P	K	pH_s_	EC_e_	WBOC	N	P	K
*Tillage*												
Conventional	7.96	4.79	3.85	85.6	23.0^B^	244.4	7.79	7.90	4.23	90.6	19.4^B^	214.4^B^
Reduced	7.90	4.54	3.98	90.9	25.0^A^	241.3	7.76	7.83	4.20	91.9	23.3^A^	226.2^A^
Zero	7.93	4.28	3.99	86.3	22.6^B^	250.2	7.76	7.18	4.44	91.8	21.0^B^	230.1^A^
SE_m_ ±	0.06	0.25	0.19	2.12	0.82	4.25	0.03	0.35	0.25	1.84	1.06	5.69
*Saline irrigation*												
100CWR	7.94	4.97^A^	3.88	89.8	23.8	241.8^B^	7.77	8.23^A^	4.30	94.4^A^	20.7^B^	222.2
80CWR	7.95	4.40^B^	3.96	87.2	24.0	240.7^B^	7.78	7.65^B^	4.20	89.9^B^	20.0^B^	220.5
60CWR	7.91	4.24^B^	3.99	85.9	22.8	255.2^A^	7.77	7.04^C^	4.37	90.0^B^	23.1^A^	227.7
SE_m_ ±	0.04	0.18	0.15	1.62	0.65	5.39	0.04	0.23	0.16	1.66	1.02	5.01
*Rice straw mulch*												
No mulch	7.92	4.54	3.88	85.2^B^	23.2	241.4	7.77	7.92^A^	4.17	88.1^B^	20.6	218.2^B^
Mulch	7.94	4.53	4.01	90.0^A^***	23.9	249.3	7.77	7.35^B^	4.41	94.8^A^***	21.9	229.3^A^
SE_m_ ±	0.03	0.15	0.12	1.32	0.53	4.40	0.03	0.19	0.13	1.36	0.83	4.09

Different uppercase letters (A, B) denote significant differences (** *P* < 0.01, *** *P* < 0.001, Tukey's HSD test). Data are means over 2 years. CWR: Percent water requirement for wheat, irrigation applied only in wheat, sorghum was grown as rainfed; SE_m_ ± : standard error.

**Table 2:** Analysis of variance of tillage, mulch and saline irrigation on pH_s_, EC_e_ , Walkley and Black oxidizable organic C (WBOC) and KMnO_4 _oxidizable N, Olsen’s P and NH_4_OAc extractable K , soil microbial biomass carbon and nitrogen (MBC and MBN), microbial biomass carbon nitrogen ratio (MBCN), dehydrogenase (DHA), alkaline phosphatase (AlK), Urease (Ur), *α*- and *β*-glucosidase (*α*-/*β* glu) and on green fodder yield (GFY) and dry fodder sorghum yield (DFY) and weighted linear soil health index (SHI_RS_); * *P* < 0.05, ** *P* < 0.01, *** *P* < 0.001 and NS: not significant (*P* > 0.05).

Source	DF	pH_s_	EC_e_	OC	N	P	K	MBC	MBN	MBCN	DHA	ALP	Ur	*α*-glu	*β*-glu	SHI_RS_	GFY	DFY
Replication	2	*	NS	NS	NS	NS	NS	NS	NS	NS	NS	NS	NS	*	NS	NS	NS	NS
Year	1	NS	***	NS	NS	NS	NS	NS	***	*	*	NS	**	***	NS	*	NS	NS
Tillage	2	NS	NS	NS	NS	*	NS	NS	**	*	*	*	NS	NS	NS	*	NS	NS
Year × tillage	2	NS	NS	NS	NS	NS	NS	NS	NS	NS	***	NS	NS	*	NS	*	NS	NS
Error (a)	10																	
Irrigation	2	NS	***	NS	NS	NS	*	***	**	*	***	*	NS	NS	NS	*	NS	NS
Mulch	1	NS	NS	NS	***	NS	NS	NS	**	NS	***	**	NS	***	*	*	NS	NS
Irrigation × mulch	2	NS	NS	NS	NS	NS	NS	NS	NS	NS	NS	***	NS	**	NS	NS	NS	NS
Year × irrigation	2	NS	NS	NS	**	NS	NS	*	NS	NS	NS	NS	NS	NS	NS	NS	NS	NS
Tillage × irrigation	4	NS	NS	NS	NS	NS	NS	NS	NS	NS	NS	**	*	***	NS	*	NS	NS
Year × tillage × irrigation	4	NS	NS	NS	NS	NS	**	NS	NS	NS	*	NS	NS	NS	NS	NS	NS	NS
Year × mulch	1	NS	NS	NS	NS	NS	NS	NS	NS	NS	NS	*	NS	NS	NS	NS	NS	*
Tillage × mulch	2	NS	NS	NS	NS	NS	NS	*	NS	*	NS	**	*	***	NS	*	NS	NS
Year × tillage × mulch	2	NS	NS	NS	*	NS	NS	NS	NS	NS	**	NS	*	*	NS	NS	*	**
Year × irrigation × mulch	2	NS	NS	NS	NS	NS	**	NS	NS	NS	NS	NS	NS	NS	NS	NS	*	NS
Tillage × irrigation × mulch	4	NS	NS	NS	*	NS	NS	NS	NS	NS	NS	NS	NS	NS	NS	*	*	NS
Year × tillage × irrigation × mulch	4	NS	NS	NS	NS	NS	NS	NS	NS	NS	NS	NS	NS	*	NS	NS	NS	NS

**Table 3:** Analysis of variance of tillage, mulch and saline irrigation on pH_s_, EC_e ,_ Walkley and Black oxidizable organic C (WBOC) and KMnO_4 _oxidizable N, Olsen’s P and NH_4_OAc extractable K, soil microbial biomass carbon and nitrogen (MBC and MBN), microbial biomass carbon nitrogen ratio (MBCN), dehydrogenase (DHA), alkaline phosphatase (AlK), urease (Ur), *α*- and *β*-glucosidase (*α*-/*β* glu), wheat grain and straw yield and weighted linear soil health index (SHI_IW_); * *P* < 0.05, ** *P* < 0.01, *** *P* < 0.001 and NS: not significant (*P* > 0.05).

Source	DF	pH_s_	EC_e_	OC	N	P	K	MBC	MBN	MBCN	DHA	AlP	URE	*α*-glu	*β*-glu	SHI_IW_	Grain	Straw
Replication	2	NS	NS	NS	NS	NS	NS	*	NS	*	NS	NS	NS	NS	NS	NS	NS	NS
Year	1	NS	***	NS	NS	***	**	NS	**	**	NS	NS	*	NS	NS	*	NS	*
Tillage	2	NS	NS	NS	NS	*	*	NS	**	NS	NS	NS	NS	NS	NS	*	NS	*
Year × tillage	2	NS	NS	NS	NS	NS	NS	NS	***	NS	NS	NS	NS	NS	NS	NS	NS	NS
Error (a)	10																	
Irrigation	2	NS	***	NS	*	**	NS	**	*	NS	**	**	NS	NS	NS	*	NS	NS
Mulch	1	NS	**	NS	***	NS	*	NS	NS	NS	***	***	***	NS	NS	*	NS	*
Irrigation × mulch	2	*	NS	NS	NS	NS	NS	NS	NS	NS	NS	***	NS	NS	NS	NS	NS	NS
Year × irrigation	2	NS	NS	NS	NS	NS	NS	NS	NS	NS	NS	NS	NS	NS	NS	NS	NS	NS
Tillage × irrigation	4	NS	NS	NS	*	NS	NS	NS	NS	NS	NS	NS	*	NS	NS	NS	NS	NS
Year × tillage × irrigation	4	NS	NS	NS	NS	NS	**	NS	NS	NS	NS	*	**	NS	NS	NS	NS	NS
Year × mulch	1	NS	NS	NS	NS	*	NS	NS	NS	NS	NS	NS	NS	NS	NS	NS	NS	NS
Tillage × mulch	2	NS	NS	NS	NS	NS	NS	NS	**	NS	NS	**	NS	NS	NS	NS	NS	NS
Year × tillage × mulch	2	NS	NS	NS	NS	NS	NS	NS	NS	*	NS	NS	***	NS	NS	NS	NS	NS
Year × irrigation × mulch	2	NS	NS	NS	NS	NS	NS	NS	NS	NS	NS	*	NS	NS	NS	NS	NS	NS
Tillage × irrigation × mulch	4	NS	NS	NS	*	**	NS	NS	NS	NS	NS	NS	NS	NS	NS	NS	NS	NS
Year × tillage × irrigation × mulch	4	NS	NS	NS	NS	NS	*	NS	NS	NS	NS	NS	NS	NS	NS	NS	NS	NS

### Nutrient availability and soil organic C

Rice straw mulch improved KMnO_4 _oxidizable N (*P* < 0.05; Table 1) in both the crops. Irrigation effect on KMnO_4_-N was apparent only for wheat, and 100CWR maintained higher values of KMnO_4_-N compared to deficit irrigation. The tillage × irrigation and tillage × irrigation × mulch interactions were significant (*P* < 0.05; Tables 2, 3; Supplementary Fig. 1 and 2). The RT + 100CWR had higher values of KMnO_4_-N during wheat (Supplementary Fig. 1). Irrigation equivalent to 100CWR with mulch in RT increased KMnO_4_-N in both the crops. However, CT + 100CWR + mulch and ZT + 60CWR + mulch recorded greater values of KMnO_4_-N during wheat only. The Olsen’s P increased in the second rotation (24.1 kg ha^−1^; *P* < 0.001) compared to 18.4 kg ha^−1^ in the first rotation during wheat but remained unaffected in sorghum (Supplementary Table 1)*.* The RT had greater Olsen’s P compared to CT and ZT (Table 1). The deficit irrigation (60CWR) showed higher Olsen’s P in wheat. Deficit irrigation with mulching had lower Olsen’s P across all the tillage except ZT + 60CWR and CT + 100CWR (Supplementary Fig. 2) (*P* < 0.05). Ammonium acetate (NH_4_OAc) extractable K declined after wheat harvest in the second rotation compared to the first rotation (Supplementary Table 1). Tillage effect was apparent in wheat only, and ZT and RT showed higher NH_4_OAc-K compared to CT. Mulching and deficit saline-water irrigation with 60CWR showed higher NH_4_OAcK compared to 100 and 80CWR. But, different management practices did not affect Walkley and Black oxidizable organic C (WBOC) (Table 1).

### Microbial biomass C and N

**Figure 1:**
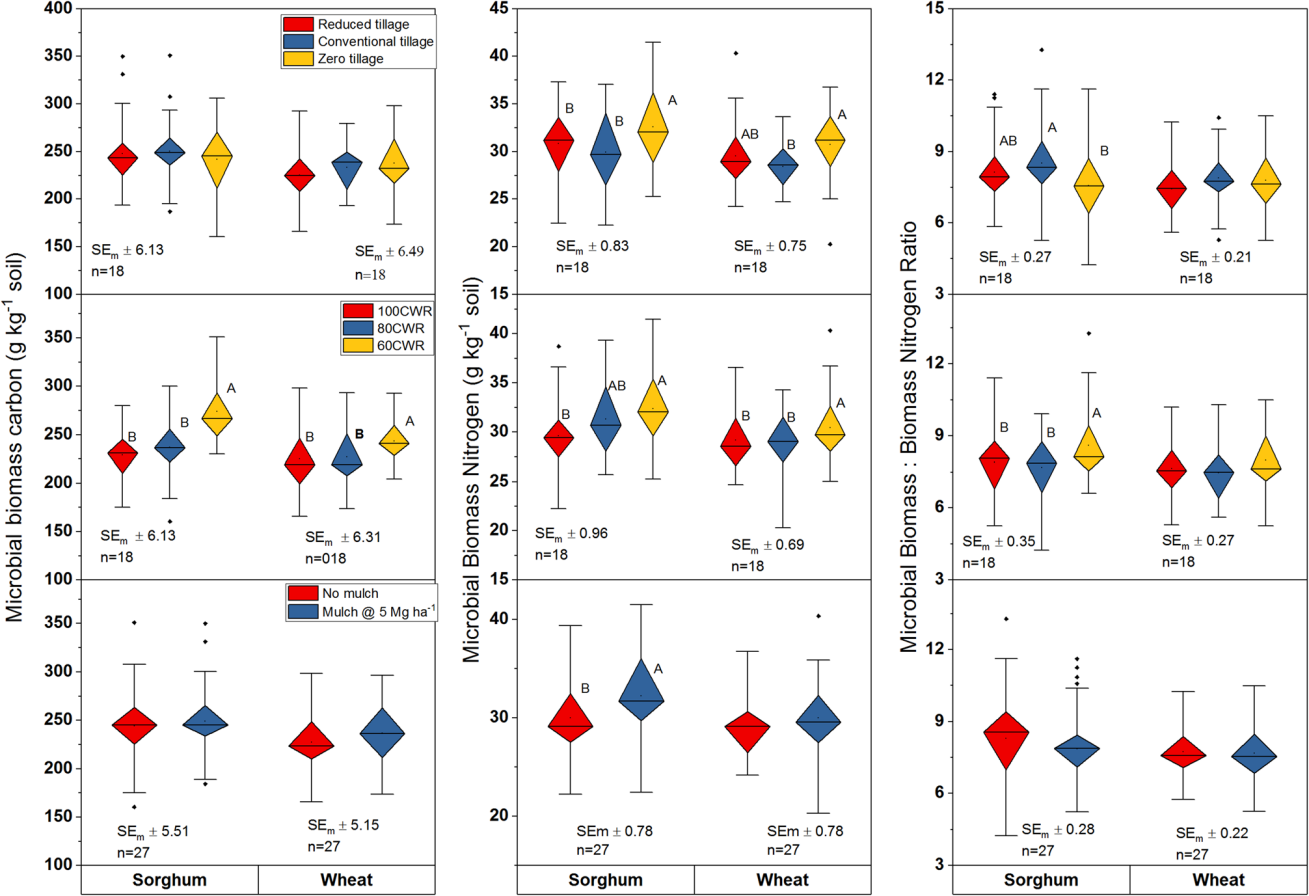
Influence of tillage, irrigation and mulch on dehydrogenase, alkaline phosphatase and urease activities; different uppercase letters (A, B) denote significant differences (**P < 0.01, ***P < 0.001, Tukey's HSD test). Data are means over 2 years. Irrigation applied only in wheat, sorghum was grown as rainfed; CWR: per cent water requirement for wheat; SE_m_ ± : standard error.

Soil microbial biomass C (MBC) in soils declined in wheat compared to sorghum. Deficit irrigation (60CWR) maintained greater values of MBC (*P* < 0.01) compared to 100CWR in both the crops (Fig. 1). The RT and mulch interaction showed greater values of MBC in sorghum (Supplementary Fig. 3). Microbial biomass N in both the crops was greater in the second rotation compared to the first rotation (Supplementary Table 1). Deficit irrigation and skipping tillage favored for higher MBN in both the crops (Fig. 1). The ZT and deficit saline-water (60CWR) showed greater values of MBN (*P* < 0.01) compared to CT. The application of mulch increased the MBN in sorghum. Tillage × mulch interaction increased the MBN in wheat. Mulch with RT showed a greater value of MBN compared with no mulch (Table 3; *P* < 0.05); but the mulching effect was not perceptible with CT and ZT. Among tillage, ZT showed a lower MBC:MBN ratio (MBCN) compared to CT in sorghum. The MBCN ratio was narrower for 100 and 80CWR compared to 60CWR in sorghum. The MBCN in CT with no mulch was higher compared to RT with no mulch and ZT with mulch in sorghum (Supplementary Fig. 3, *P* < 0.01).

### Soil enzymes activity

**Figure 2:**
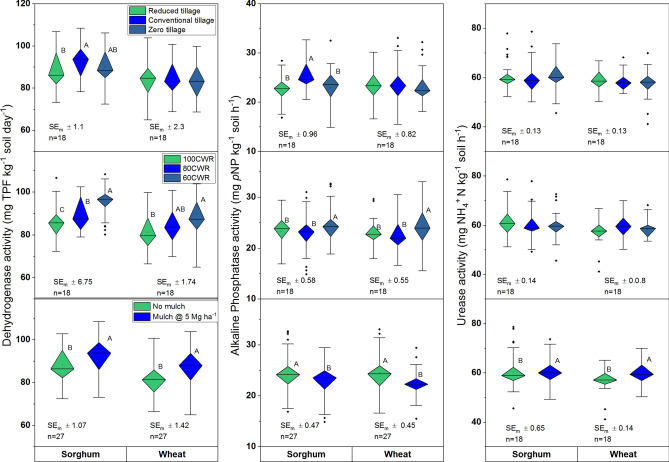
Influence of tillage, irrigation and mulch on microbial biomass carbon, nitrogen and carbon and nitrogen ratio (MBCN) of soils after sorghum and wheat harvest; different uppercase letters (A, B) denote significant differences (** *P* < 0.01, *** *P* < 0.001, Tukey's HSD test). Data are means over 2 years. Irrigation applied only in wheat, sorghum was grown as rainfed; CWR: per cent water requirement for wheat; SE_m_ ± : standard error.

Dehydrogenase activity (DHA) increased in sorghum after the second rotation (Supplementary Table 1). The CT improved DHA activity compared to RT and ZT in sorghum (Fig. 2). Further, 60CWR showed greater DHA compared to 100 and 80CWR in both the crops. Mulching also increased DHA compared to no mulch. CT showed greater values of alkaline phosphatase activity (AlP) than ZT and RT in sorghum (Fig. 2). Application of 60CWR and no mulch had higher values of AlP compared to 100CWR/80CWR and mulch, respectively, in both the crops. The saline-water irrigation × mulch interaction showed that 60CWR with no mulch had a higher value of AlP in sorghum (Table 2; Supplementary Fig. 4). The tillage × saline irrigation and tillage × mulch interactions were also significant in sorghum. The CT with 60CWR had greater values of AlP in sorghum (Supplementary Fig. 1). While CT with no mulch showed greater AlP in sorghum than CT + mulch and RT and ZT (Supplementary Fig. 4). Urease (Ur) activity was similar in different tillage and irrigation treatments. However, mulching favored the greater Ur activity in both the crops (Fig. 2). The ZT with 100CWR showed greater values of Ur in sorghum soils, while, 100CWR declined Ur activity in wheat in CT and ZT. The ZT + mulch also had greater Ur in sorghum.

The *β*-glucosdase activity (*β*-glu) was greater than *α*-glucosidase (*α*-glu) (Supplementary Table 2). Both the glucosidases were unaffected by tillage and irrigation management. Mulching increased the activities of *β*-glu and *α*-glu in sorghum only. The tillage, irrigation and mulch interaction on *α*-glu was not perceptible in wheat. Mulch with 60CWR had higher values of *α*-glu in sorghum (Table 2; Supplementary Fig. 4); while, 60CWR in RT increased *α*-glu activity in sorghum soil. The RT + mulch showed greater values of *α*-glu than CT and ZT in sorghum.

### Sorghum and wheat yield

Green and dry fodder yield of sorghum were at par for different tillage, mulch and irrigation practices (Table 4). However, deficit saline irrigation at 60CWR with mulch in RT showed higher green fodder yield (Table 2; Supplementary Fig. 2). The grain yield of wheat was increased by 4.3% in the second rotation (6.04 Mg ha^−1^; *P* < 0.05) compared to the first rotation (5.78 Mg ha^−1^). Grain yield was reduced under ZT compared to CT, while, in RT grain yield was at par with CT. Grain yield of wheat improved by 4.6% in mulch compared to no mulch.

**Table 4:** Effect of tillage, irrigation and mulch on green fodder yield (GFY) and dry fodder sorghum yield (DFY) and wheat grain and straw yield (Mg ha^−1^) and weighted linear soil health index (SHI).

Treatments	Sorghum			Wheat		
	GFY	DFY	SHI_RS_	Grain	Straw	SHI_IW_
*Tillage*						
Conventional	53.7	12.7	0.50^B^	6.10^A^	8.95	0.40^B^
Reduced	55.8	14.0	0.51^AB^	5.94^AB^	8.73	0.41^A^
Zero	56.5	14.1	0.52^A^	5.70^B^	8.01	0.41^A^
SE_m_ ±	4.58	1.21	0.01	0.12	0.40	
*Saline irrigation*						
100CWR	53.5	13.2	0.49^C^	5.86	8.36	0.40^B^
80CWR	54.7	13.6	0.51^B^	5.98	8.91	0.40^B^
60CWR	57.6	14.0	0.53^A^	5.90	8.42	0.42^A^
SE_m_ ±	1.94	0.51	0.01	0.13	0.27	0.01
*Mulch*						
No mulch	54.7	13.3	0.49^B^	5.77^B^	8.54	0.40^B^
Mulch	55.9	13.9	0.53^A^	6.05^A^	8.59	0.39^B^
SE_m_ ±	1.58	0.42	0.01	0.11	0.22	0.42^A^

Different uppercase letters (A, B) denote significant differences (*P* < 0.05, Tukey's HSD test). Data are means over 2 years. CWR: Per cent water requirement for wheat, irrigation applied only in wheat, sorghum was grown as rainfed; SE_m_ ± : standard error.

SHI for sorghum (SHI_RS_) = ∑ [(*α*-glu score × 0.1) + (MBC score × 0.1) + (EC_e_ score × 0.09) + (KMnO_4_-N score × 0.09) + (MBN score × 0.07) + (MBCN ratio score × 0.07)].

SHI for wheat (SHI_IW_) = ∑ [(MBC:MBN ratio score × 0.11) + (EC_e_ score × 0.11) + (MBC score × 0.11) + (Olsen’s P score × 0.11) + (Urease score × 0.08) + (KMnO_4_-N score × 0.06)].

### Soil health indicators

**Figure 3:**
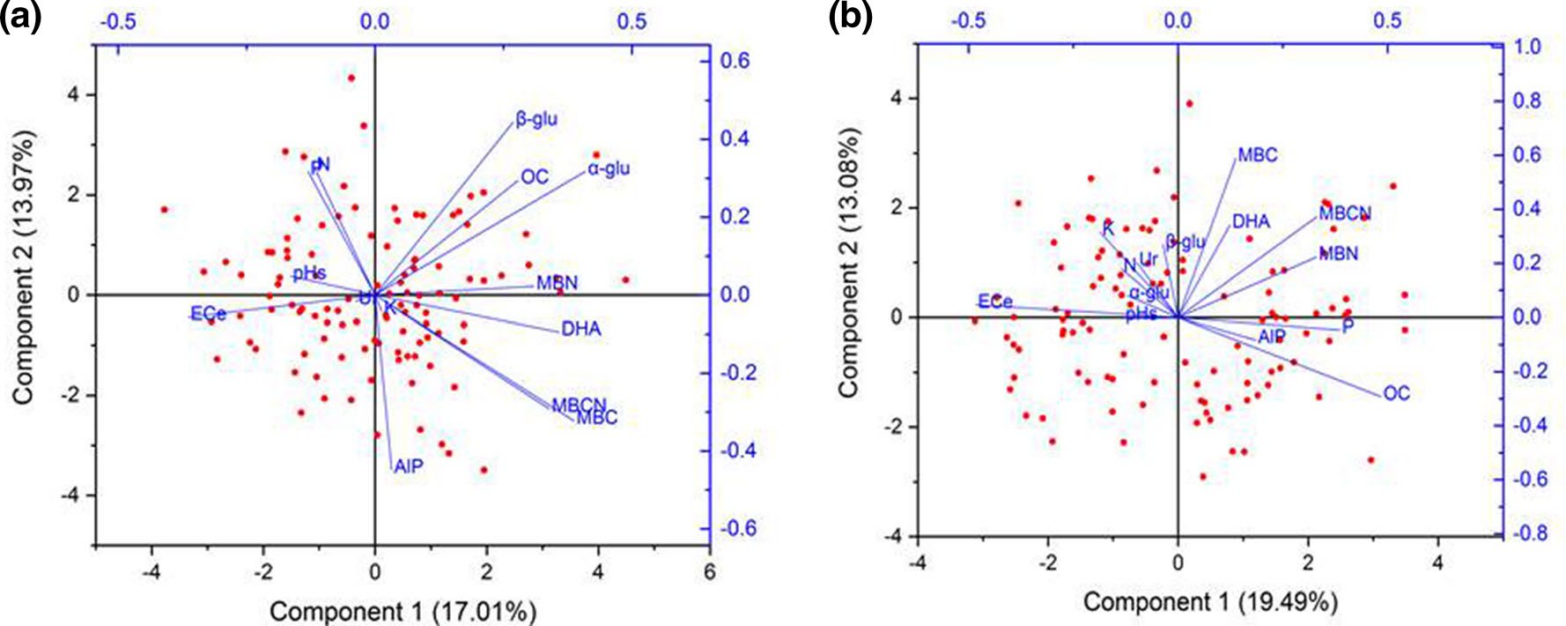
Graphic biplot for principal components (PC1 and PC2) for soil properties after harvest of sorghum (**a**) and wheat (**b**) [EC_e_, electrical conductivity of soil water saturation paste extract; pH_s_, pH of soil water saturation paste; WBOC, Walkley and Black organic carbon; MBC, microbial biomass carbon; MBN, microbial biomass nitrogen; MBCN, microbial biomass carbon to nitrogen ratio; DHA, dehydrogenase; AlK, alkaline phosphatase; Ur, urease; *α*-glu, *α*-glucosidase; *β*-glu, *β*-glucosidase].

In sorghum, the microbial biomass C showed a significant positive correlation with green fodder yield (*P* < 0.05). The electrical conductivity (EC_e_) had a negative correlation with MBN, DHA and *β*-glu (see Supplementary Table 3). First six principal components (PCs) extracted with eigenvalues > 0.99 explained 67% of the variances (Fig. 3a; Supplementary Table 5). The *α*-glu with highest loading (0.41) in PC1 and MBC (0.39) with loading within 10% of the highest weight of *α*-glu were selected. Similarly, KMnO_4_-N with the highest loading (− 0.44) in PC2 and EC_e_ (0.44) with loading within 10% of the highest weight was screened from PC2. MBN and MBC:MBN ratio were screened from PC3 and PC4, respectively. In wheat, the first six PCs (eigenvalues > 1.0) explained 62% of the variances (Fig. 3b; For correlation matrix see Supplementary Table 4; See Supplementary Table 6). The MBC:MBN ratio, Olsen’s P (0.41) and EC_e_ (− 0.42) were selected in PC1. Similarly, MBC, Ur, and KMnO_4_-N were screened from PC2, PC3, and PC4, respectively. No attributes were selected from PC5 and PC6 in both the crops_._

### Indicator interpretation and development of soil health index

A normalized scale of 0 to 1 by using linear scoring functions used to screen indicators selected in PCA for the soil in both the crops. For sorghum, the weighted factors 0.10 for both *α*-glu and MBC from PC1, 0.09 for both KMnO_4_-N and EC_e_ from PC2, 0.07 for MBN and MBC:MBN ratio from PC3 and PC4, respectively, were used (Table 2). The weighted minimum data set (MDS) indicator scores summed up for each observation for obtaining a soil health index (SHI_RS_). Similarly, for wheat, a weighted factor of 0.11 for MBC:MBN ratio, EC_e,_ and Olsen’s P from PC1, 0.11 for MBC from PC2, 0.08 for Ur from PC3, 0.06 for KMnO_4_-N from PC4 were used to obtain soil health index (SHI_IW_).

Cultivation improved SHI for soil with every crop cycle (*P* < 0.01; Supplementary Table 1^1^. The ZT and RT improved soil health compared to CT (Table 4) in both the crops. Deficit saline irrigation (60CWR) and mulching showed higher values of SHI in sorghum and wheat. Sorghum dry fodder yield was significantly correlated to SHI_RS_ (*P* < 0.05, *R*^2^ = 0.30) and explained the variability in dry fodder yield (Supplementary Fig. 5). Oppositely, SHI_IW_ and wheat grain yield had no relation (*R*^2^ = 0.03).

## Discussion

Irrigated agriculture is under the pressure of meeting the challenges of the Sustainable Development Goals (SDGs) related to food, water and health^21,22^. The productive utilization of the available saline-water resources can reduce the growing pressure on the natural resources important for food security^23^. Soil salinization because of inadequate water management is widespread in the semi-arid part of the world^24^. In the present study, the salinity developed in surface soil was the resultant of the salt addition through saline-water irrigation and cyclic downward and upward salt flux in different seasons. Therefore, soil after harvest of wheat showed higher EC_e_ compared to sorghum because of saline-water irrigation and upward flux of salt^25^. Mulch was effective in reducing salt in surface soil in both seasons because of hindered evaporation and upward salt flux in surface soil^25^. Effective use of the saline soil–water resources needs a system approach in devising nature-based solutions utilizing its characteristics and dynamics^26^. The declining trend in EC_e_ with every crop cycle pointed to lesser risk of salinity development by adopting deficit saline-water irrigation and mulching in rainfed sorghum-irrigated wheat cropping system. The higher rainfall in the second year (634 mm) compared to first-year (524 mm) also favoured a gradual decline in salinity with time. Other research results also establish that more than 80% of the salts accumulated in saline-water irrigated wheat gets leached with an annual rainfall exceeding 500 mm^27,28^. The rainfed sorghum in monsoon season acted as a balancing feedback loop to make the production system resilient by leaching out the salt accumulated by saline-water irrigation in wheat^29^.

Mulching improved the KMnO_4_-N, Olsen’s P and NH_4_OAc-K of the soil in both crop seasons. The surface applied mulch helped in producing favorable conditions for soil microbial activity during dry spell was responsible for organic matter decomposition and mineralization of N, P and K associated with soil organic matter^25,30^. Further, decomposition of organic mulch also promoted natural leaching of soluble salts from the root zone, mineralization and release of nutrients^31^. Application of organic residues and mulching normally increases the N mineralization potential of soils, responsible for, an increase in KMnO_4_-N^32,33^. Increased DHA, Ur and *α*/*β-*glu activities in mulching also pointed to the greater soil metabolic activity responsible for organic N mineralization^34^. Although mulching improved the KMnO_4_-N, it was decreased by mulching in deficit irrigation treatments. As a standard agronomic practice, about 50% of total nitrogenous fertilizers are broadcast in standing crop after application of straw mulch. A part of the applied fertilizer holds onto the straw and subsequently lost through volatilization during the drying period. In deficit irrigation treatments, surface retained N is more liable to losses because of less water available for eluting the N fertilizers retained on mulch.

Olsen’s P was the same in both rotations because of an external supply of fertilizer P and mineralization of organic P over time^35,36^. Tillage effect was apparent on Olsen’s P, and RT showed a higher values of Olsen’s P, which can be attributed to increased immobilization of P by soil microorganisms^37^ in CT and ZT. The reduced tillage had been reported to offer 1.7 times improvements in P availability in oxisols^37^. Changes in Olsen’s P status of these soils were attributed to change in soil aggregation and P fixation under different tillage^37,38^. Low availability of P in ZT may also be attributed to increased leaching of dissolved reactive P through macropores’ developed under these practices^39^. In CT, increased P fixation because of the uniform mixing of P fertilizers in plough layer might be responsible for low Olsen’s P.

The interaction between mulch, irrigation and tillage also varied with nutrients. Deficit irrigation and mulching interaction caused an increase in Olsen’s P, while, a decrease in KMnO_4_-N content. The observed variation in availability pattern might be because of the difference in the method of soil application. All the P fertilizers were applied at sowing, hence immobility of P and less water availability for leaching favored for higher Olsen’s P in surface soil of 60CWR compared to 100CWR and 80CWR. Less salt import in 60CWR also improved the Olsen’s P because of lower salt load in soil compared to 100CWR and 80CWR altered the P dynamics by the change in P sorption sites of soil, the dominance of cations (Na^+^, Ca^2+^ and Mg^2+^) and supply of SO_4_^2−^^40^. The presence of ligand substances as supplied by mulch also facilitated the chelation of soil solution phosphorus^41–43^. Mass flow is an important mechanism of potassium transport in soil. Deficit irrigation showed a large accumulation of NH_4_OAc-K in surface soil. Less water available for leaching of unabsorbed potassium remaining in soil solution was the main reason for higher potassium in 60CWR compared to 100CWR and 80CWR. The ZT and RT also favored increased NH_4_OAc-K in surface soils because of the relocation of K from the lower layer in absence of uniform mixing of soil in ZT and RT (no-tillage in wheat season) contrary to CT^38^.

Although several studies report greater MBC in ZT and RT than CT in surface soil because of surface accumulation crop residue in ZT and RT in contrast to even distribution in the plough layer of CT^44^. In the present study, changes in MBC in different tillage were not perceptible because of shorter duration. Other groups of researchers also reported increased MBC because of the application of manures, crop residue and legumes supplying energy for microbial proliferation^44–47^. Decreased survival of microorganisms because of increased salinity and moisture stress prevailing in March–April at the experimental site was mainly responsible for lower MBC values after wheat compared to sorghum harvest. This finding conforms with others^34,48^ those recorded lower values of MBC in the soil after harvest of wheat compared to sorghum in saline soils. The 100CWR saline-water irrigation imported more salts compared to deficit saline-water irrigation (60 and 80CWR) and showed lower values of MBC. Change in salinity under deficit irrigation was negatively correlated with MBC content (*r* = − 0.21 to − 0.25). This observation reconfirmed that increment in microbial biomass C depends on osmotic stress developed because of saline-water induced salinity of surface soils^49^. The similar values of MBC in tillage and rice straw mulch in the present study might be because of the suppressive effect of osmotic stress on mulch and tillage induced changes in microbial populations^50^. This non-significant effect of mulch on MBC was in agreement with the previous studies^51^. Besides osmotic stress, decomposition of rice straw by microorganism was extremely regulated because of the high C/N ratio, lignin and polyphenols content of applied rice straw mulch^47,52^. Therefore, the impact of rice straw on MBC was somewhat different from those observed for easily decomposable organic substrates^49^. Although MBC was not affected by different management practices, however, it had a negative correlation with EC_e_ (*r* = − 0.25; − 0.32). This early trend indicated the overriding impact of EC_e_ compared to other factors affecting MBC.

The MBN was more sensitive to different tillage, irrigation and mulch treatments compared to MBC. The observed differences in sensitivity were because of reduced soil salinity under ZT, 60CWR and mulched treatments (Table 1). Changes in soil salinity affect the compositions of soil microbial community^53^. The lower EC_e_ values in ZT, 60CWR and mulch promoted the proliferation of microorganisms having more N requirement per unit carbon in their biomass. This is further accorded with observed negative correlation with EC_e_ (Supplementary Table 2 and 3) and lower MBCN (MBC/MBN ratio) in ZT and mulch treatment except for 60CWR irrigation. The MBCN denoted the dominance of specific categories of microbial population^54^. This ratio resided in the range of 7.75–8.40 and 7.38–8.03 in soils after the harvest of sorghum and wheat, respectively. The reported ratio is slightly higher than others^53^.

Measuring the activities of phosphatase, dehydrogenase, urease and glucosidase serve as an early indicator of changes in soil health of saline soil brought under cultivation^43,48^. Activities of these enzymes increased with a gradual decline in EC_e_ after every crop cycle. Enzyme activities were more sensitive to salinity stress and the availability of organic substrates. With the alleviation of salinity stress during *kharif* season, the effect of mulching was evident on all the enzymes, while the tillage effect was apparent on dehydrogenase, alkaline phosphatase and urease activities. Enhanced activity of these enzymes in CT in *kharif* season was mainly associated with the increased availability of organic substrates (crop residue and organic mulch) for soil metabolic activities. Conventional tillage uniformly mixes the entire crop residue in plough layer whereas in ZT and RT all the residue retained on the surface in the absent (ZT) or partial (RT) tillage practices. This impact of substrate availability on DHA was diminished during the *rabi* season because of high temperature and greater osmotic/matric potential during soil sampling period (April). Previous findings also claimed for a decrease in *β*-glucosidase activity by increasing salinity^43^. Olsen’s P showed negative correlation with alkaline phosphatase activity (*r* = − 0.64, *P* < 0.01). Phosphatases are adoptive enzymes, the intensity of their exudation by microbes to some extent depends on the demand for phosphorus. This was evident from increased AlP in CT having low Olsen’s P. Contrary to other biological attributes (MBC and DHA), glucosidase activity increased in wheat compared to sorghum. An appreciable amount of polysaccharides that were available from sorghum stubble and rice straw mulch^44,53^ might have increased the activity of glucosidases in wheat. Besides, the mulch applied in wheat produced a non-significant effect on glucosidases because of the low decomposition of surface applied mulch. Contrarily, mulching favored both glucosidase activities in sorghum because rice straw mulch decomposition supplied more cellulose during monsoon season^44^. Similarly, greater Ur activity because of mulching and ZT may be attributed to an increased availability of organic substrate and reduced salinity in surface soil for accounting for higher biological activity.

Soil health index provides an early indication of the soil functions for specific land use and relates management strategies to outcomes^55,56^. A different set of indicators screened for developing soil health index for sorghum and wheat in contrasting soil environments (rainfed sorghum and saline irrigated wheat) and with different management practices. Therefore, the different sets of soil health indicators screened for assessing the two crops were rational. Further, the captured soil health index (SHI) varied significantly and its values were edged over in sorghum compared to wheat. The calculated SHI varied because of differences in cultural practices (irrigation), seasonal variation in soil salinity and soil temperature during sorghum and wheat season. The increase in EC_e_ and decrease in value of soil attributes in wheat compared to sorghum showed lower values of SHI. Among the tillage, ZT showed greater SHI than CT for soils in both the crops, because of relatively decreased soil salinity in ZT compared to CT and RT. Previous studies also used different soil health indicators to assess SHI and found that management practices had a dominant influence on SHI. Besides management practices, variation in rainfall was also responsible for seasonal variation in SHI. Rainfall indirectly affects different soil attribute by its effect on the leaching of salt from surface soil. Previous results also showed that salt leaching efficiency varies with the amount of rainfall, soil texture and inherent soil–water salinity^16,57,58^. Hence, the observed effect of management strategies on SHI will need site-specific standardization to account for the spatial and temporal variation across the geographical units^59,60^.

The derived SHI was effective in predicting dry fodder yield under salt-affected soils. However, this relation was not observed between SHI and wheat yield, as the variety was salt-tolerant, and the stress effect was obliterated^61^. The inherent salinity tolerance mechanism of the cultivar was capable to overcome the stress. Besides, a change in SHI in the early stage of land conversion was not sufficient to affect crop yield. The SHI might improve further, but may or not affect crop yield of salt-tolerant variety. However, its relationship with wheat grain yield could be validated in future studies using moderate to sensitive wheat cultivars.

## Conclusions

Our study concludes that zero tillage (ZT) effectively reduced soil salinity and improved soil health. Deficit saline irrigation improved the nutrient availability (P and K), metabolic activities (microbial biomass C, microbial biomass N, dehydrogenase and alkaline phosphatase) with increased soil health. Mulching also improved the availability of N and K, increased dehydrogenase, urease and *α*/*β*-glucosidases activities. The multivariate analysis recommended *α*-glucosidase activity, MBC, EC_e_, KMnO_4 _oxidizable N and urease activity as soil health indicators. Reduced tillage with deficit saline-water irrigation and mulch promoted soil health without a decline in sorghum and wheat yield compared to conventional tillage (CT). The developed protocol for indexing soil health and screening of soil health indicators may be applicable for salt-affected areas of arid and semiarid countries with similar soil and rainfed-irrigated ecologies. This study also establishes the benefit of soil health improvement with a reduced cost of land preparation without yield decline during the transition from CT to ZT. Soil health index may improve further and long-term study may establish the actual relationship between SHI and yield of salt-tolerant varieties in salt-affected soils.

## Materials and methods

### Experimental site

**Figure 4:**
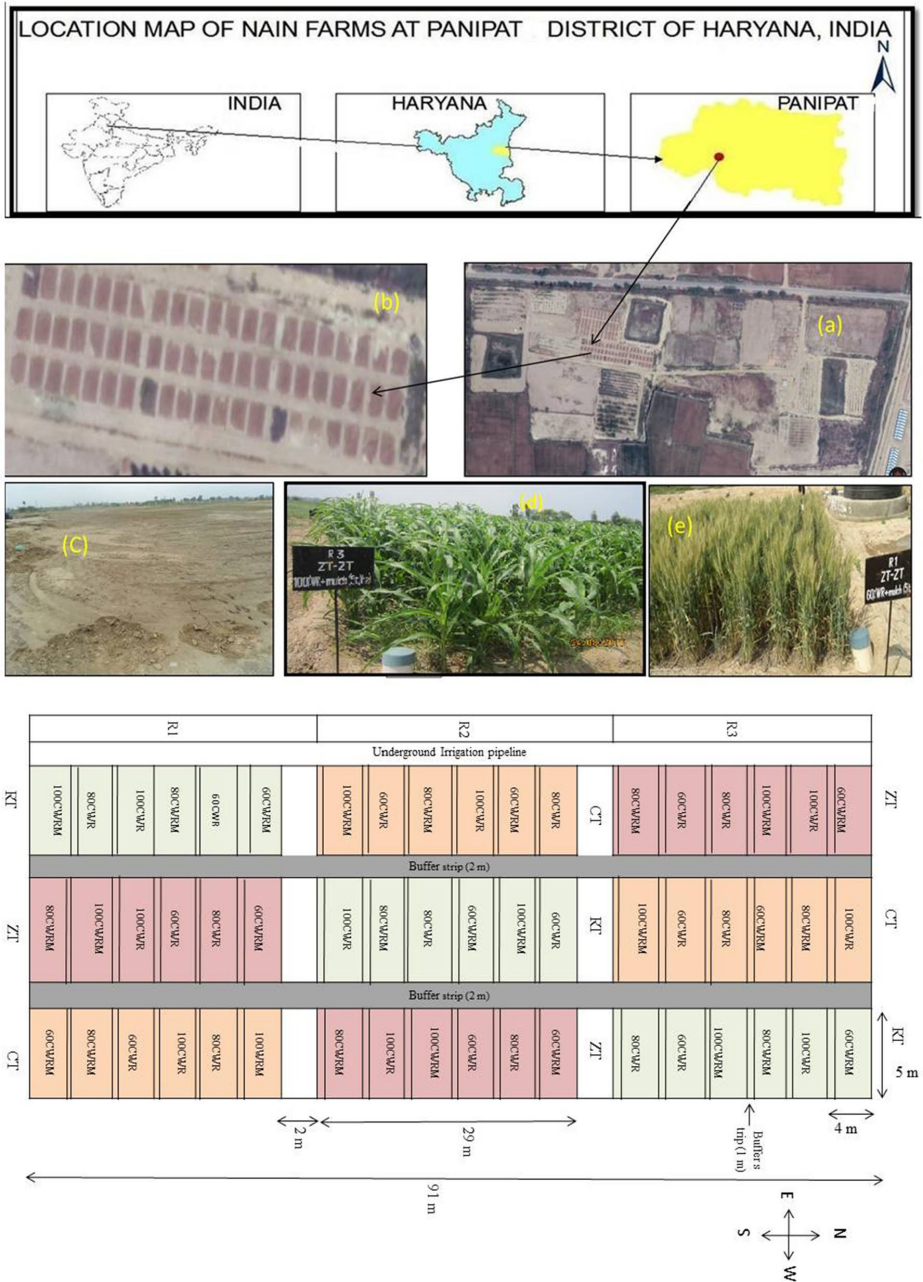
(**A**) Location map of field experiment at Panipat, Haryana, India; (a) CSSRI experimental Farm, Nain, Panipat, Haryana; (b) Experimental field; (c) Barren field before cultivation May 2014; (d) *Kharif* sorghum; (e) *Rabi* wheat; (**B**) Layout of field experiment. Main Plot: 29 m × 5 m; Sub-plot: 4 m × 5 m; between main plot 2 m buffer strip; between two sub plot-buffer strip of 1 m; ZT, zero tillage; CT, conventional tillage; RT, reduced tillage; 100, 80 and 60, per cent water requirement of wheat crop, irrigation applied only in wheat, sorghum was grown as rainfed; M, 5 Mg ha^−1^ rice straw mulch applied during wheat.

A field experiment with sorghum-wheat cropping system was conducted from July 2014 to April 2017 at the Nain experimental farm (29°19′ 7.09′′ to 29°19′10.0′′ N latitude and 76°47′30.0′′ to 76°48′0.0′′ E longitude), ICAR-Central Soil Salinity Research Institute Karnal, Haryana, India (Fig. 4a). The site was the barren saline land and brought under cultivation for the first time. The soil texture of the experimental field was sandy loam and classified under mixed *Sodic Haplustepts* (saline phase) following the USDA soil taxonomic classification system^62^. Soil samples were collected in the last week of June 2014 before the start of the experiment with pH_s_ 8.2 ± 0.2 and EC_e_ 16.2 ± 9.6 dS m^−1^ and the detailed physicochemical properties of soils is presented in Supplementary Table 7. The meteorological data recorded during the cropping season is presented in Supplementary Fig. 6.

### Field experiment

The field experiment was conducted in a split- factorial plot design with three replications. The main plot size was 29 × 5 m^2^, and the sub-plot size was 4 × 5 m^2^ (Fig. 4b). Three tillage treatments viz*.* reduced tillage (RT), conventional tillage (CT) and zero tillage (ZT) were taken in the main plot and irrigation treatments comprising of rainfed sorghum and irrigated wheat with saline-water (EC_iw_ 8.0 dS m^−1^) irrigation equivalent to 100, 80 and 60 per cent of wheat crop water requirement (CWR) and rice straw mulch (0 and 5 Mg ha^−1^) in subplots. Rice straw was applied uniformly in inter-row space at 2–3 leaf stage. After wheat harvesting, in June, remaining rice straw mulch in conventional tilled (CT) plots was plowed into the soil to a depth of 0–15 cm. Whereas, in RT and ZT plots remaining rice straw mulch of the previous season were retained on the surface.

*Kharif* (July to October) sorghum was taken as a rainfed crop received mean rain of 426.1 ± 90.8 mm. The Wheat crop was irrigated by the surface flooding method in the *rabi* season (November to April). At the experimental site, the shallow aquifer (40 m below ground level) with lower yield was having an electrical conductivity of 3.0 dS m^−1^ while the deeper aquifer (80 m below ground level) with high yield was having high salinity (16.0 dS m^−1^). The saline-water (EC_iw_ 8.0 dS m^−1^) used for irrigation of wheat crop was prepared by mixing these two sources of groundwater at a definite proportion in 5000.0 L high-density polyethylene tank. The composition of irrigation water is described in Supplementary Table 8. Wheat is sensitive to moisture stress at crown root initiation, tillering, late jointing, flowering and dough stages. In a previous study, a threshold salinity of 8.0 dS m^−1^ for wheat was also established when saline-water irrigation was applied in light-textured soil^9^.

Further, germination and early establishment of wheat in saline soils are also sensitive to salt stress^51^. In these arid and semi-arid areas, 7–8 cm water equivalent to one irrigation are available either from rainwater harvested in farm ponds or floating best quality water of low salinity in shallow aquifers. Considering these facts, it was decided to apply pre-sowing irrigation of 7.0 cm with BAW (3.0 dS m^−1^) in all the treatments to reduce osmotic stress during germination and early growth stage. The wheat crop requires about 35.0 cm of irrigation water applied at five critical stages each of 7.0 cm depth^9^. Irrigation treatment started with first irrigation at the crown root initiation (CRI) stage (21 DAS) and continued to fifth irrigation at the dough stage (110–115 DAS). Irrigation water was applied using flood irrigation via. a 7.5 cm plastic hose fitted with a flow meter to record the amount of water used in each plot. At each irrigation, 1.4, 1.12 and 0.84 m^3^ per plot water was applied in 100, 80 and 60CWR treatments, respectively. No irrigation water was applied during the sorghum growing season.

Under CT treatments the field was prepared by one ploughing and one harrowing of 15 cm depth followed by planking in both season; while under RT, only one harrowing followed by planking was applied in *kharif* season only. In ZT, sowing was performed by a zero-till seed drill without any preparatory tillage. The recommended dose of NPK (kg ha^−1^) was applied to sorghum (120:40:30) and wheat (150:60:30). For sorghum, half of the nitrogen and a full dose of P and K were applied at sowing and the remaining nitrogen was applied 30–45 days after sowing on receipt of rainfall (54.0 ± 19.8 mm) in the month of August. For wheat crop, half of the nitrogen and a full dose of P and K were applied at sowing, and the remaining nitrogen was applied in two equal splits at first and second irrigation. Sorghum was harvested as fodder and wheat for straw and grain yield in October and April, respectively.

### Soil analyses

Plot-wise soil samples were collected from surface soil (0–15 cm) of all plots after the harvest of each crop during the second and third rotation (sorghum October 2015 and 2016; wheat April 2016 and 2017). Immediately after collection, a part of fresh soil samples after removing coarse material and roots were stored at 4˚C for estimating microbiological attributes. Further, a part of the soil was air-dried, ground using a wooden pestle and mortar to pass through a 2-mm sieve, and analyzed for soil chemical attributes.

The soil pH_s_ was determined in aqueous soil paste of the soil and water by using a digital pH meter^63^. For the determination of electrical conductivity of saturation extract (EC_e_), the aqueous extract of saturated soil paste was readily removed from the soil paste under suction at 0.88 kg cm^−2^ force^63^. Walkley and Black oxidizable organic C (WBOC), KMnO_4 _oxidizable N, Olsen’s P and NH_4_OAc extractableK were estimated following chromic acid wet oxidation followed by acidified standard FeSO_4_·(NH4)_2_SO_4_·6H_2_O titration for WBOC^64^; alkaline potassium permanganate distillation method^65^ for KMnO_4_-N; Olsen's extractant (0.5 *M* NaHCO_3_) followed by ascorbic acid reductant method for Olsen’s-P and flame photometer using neutral normal NH_4_OAc extractant^64^ for NH_4_OAc-K, respectively. The microbiological attributes, such as microbial biomass C and N (MBC and MBN) were determined in the stored samples following chloroform fumigation and extraction with 0.5 *M* K_2_SO_4_ methods^66^; Microbial biomass C flush calculated using the relationship: MBC = ((1/0.38) × C-flush)^66^; Both fumigated and non-fumigated microbial biomass N were extracted with 0.5* M* K_2_SO_4_. Distillation was carried out to find the nitrogen content. The difference in N between the fumigated and non-fumigated samples divided by a calibration factor (K_EC_) 0.38 gave the measure of MBN in soil^67^. Soil enzymes viz., dehydrogenase (DHA) activity by triphenylformazan production followed by measurement of the intensity of red colour at 485 nm wavelength^68^; for urease (Ur) activity determination, NH_4_-N released was estimated by steam distillation of an aliquot of the resulting soil suspension with MgO when 5.0 g of soil was incubated with 9 ml of 0.05 *M* THAM buffer (pH 9.0) and 0.2 *M* of urea solution at 37 °C for 2.0 h^68^. The *α* and *β*-glucosidase (*α* and *β*-glu), and alkaline (AlP, pH 11.0) phosphatase activities measured by estimating the concentration of *p*-nitrophenol released on incubation of soil with respective substrate: *α* and *β*-glucopyranoside and *p*-nitrophenyl phosphate^68^.

### Minimum data set (MDS), indicator interpretation and soil health index (SHI) development

A comprehensive soil health index was developed for sorghum and wheat separately following the method suggested by Andrew and Carrol and Mandal et al.^69,70^. The PCs with eigenvalues ≥ 1 and explained > 5% of the variance in the total dataset was only considered. Within the same PC, only highly weighted factors were retained for the MDS. The indicators were interpreted by transforming MDS by weighted linear scoring function as suggested by Andrew and Carrol^69^. After transforming, the MDS variables were provided weightage to get the soil health index (SHI) value as mentioned in Eq. (1).

1 $${{\rm SHI}} = \mathop \sum \limits_{{{{\rm i}} = 1}}^{{{\rm n}}} {{\rm wi }} \times {{\rm si}}$$

where s = soil health indicator score, w = principal components weightage factor.

### Statistical analyses

The data recorded for different crop parameters were analyzed using the analysis of variance (ANOVA) technique for a split-factorial plot design using a SAS macro (http://sscnars.icar.gov.in/spltfactm2s2.aspx). The summary of the ANOVA table is presented in Tables 2 and 3. Some parameters showed the year-wise variation hence it was also taken as a variable for analysis of variance. A pair-wise comparison of the effect of the treatments was performed using Tukey’s test at *P* ≤ 0.05. A comparison of the means for interaction effects was made using the least significant difference (LSD at *P* ≤ 0.05).

## Supplementary Information

Supplementary Information.

## Data Availability

The data supporting the findings in the manuscript is available from the corresponding author on a reasonable request.
