# Author Correction: Deficit saline water irrigation under reduced tillage and residue mulch improves soil health in sorghum-wheat cropping system in semi-arid region

**DOI:** 10.1038/s41598-021-88338-w

**Published:** 2021-04-19

**Authors:** Pooja Gupta Soni, Nirmalendu Basak, Arvind Kumar Rai, Parul Sundha, Bhaskar Narjary, Parveen Kumar, Gajender Yadav, Satyendra Kumar, Rajender Kumar Yadav

**Affiliations:** 1 ICAR-Central Soil Salinity Research Institute, Karnal, Haryana 132 001 India; 2 IARI-Krishi Vigyan Kendra, Shikohpur, Gurugram, Gurugram, Haryana 122 004 India

Correction to: *Scientific Reports* 10.1038/s41598-020-80364-4, published online 21 January 2021

The original version of this Article contained an error in the order of the Figures. Figures 1 and 2 were published as Figures 2 and 1. The Figure legends were correct.

The original Figures 1 and 2 and accompanying legends appear below.

**Figure 1:**
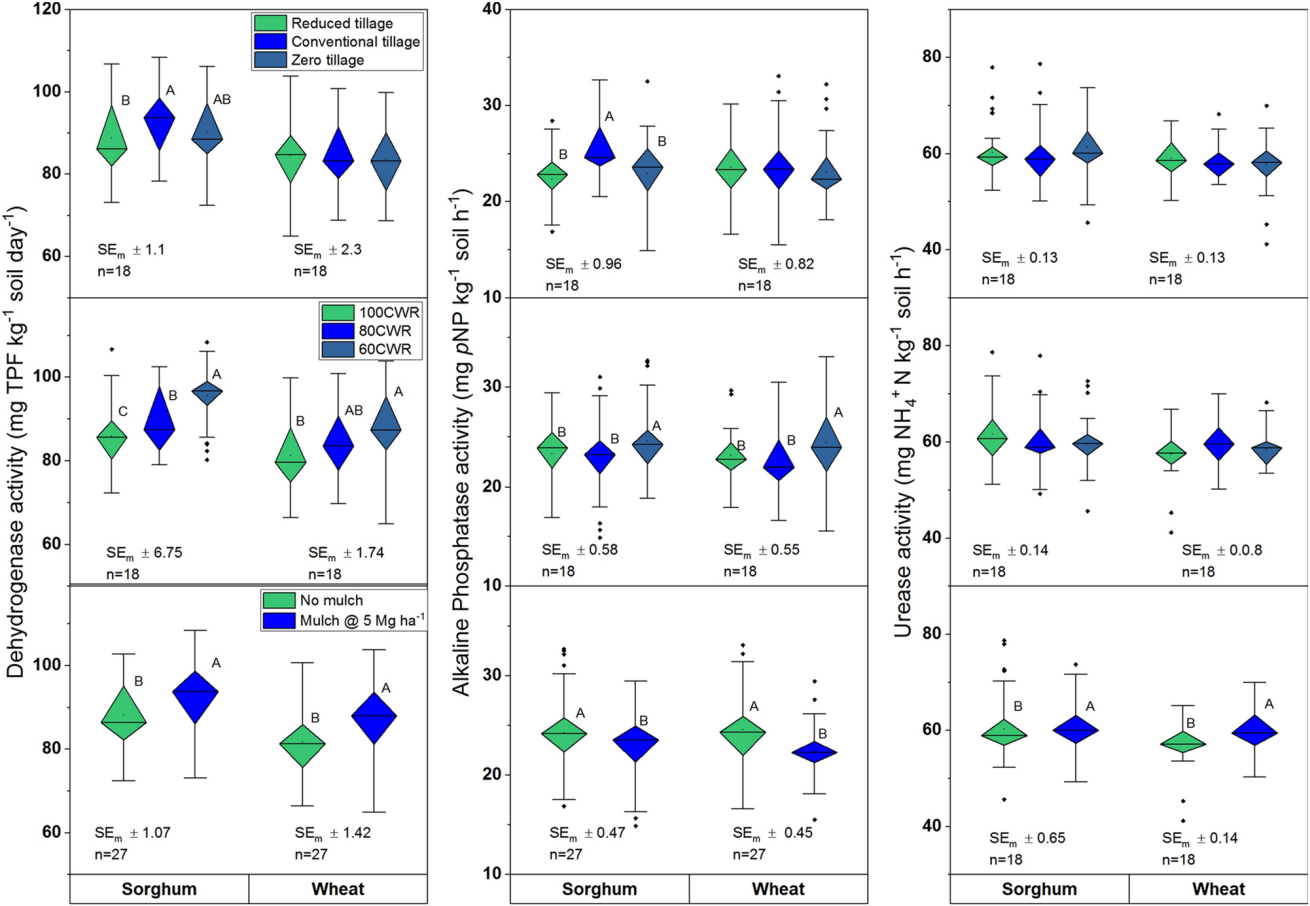
Influence of tillage, irrigation and mulch on microbial biomass carbon, nitrogen and carbon and nitrogen ratio (MBCN) of soils after sorghum and wheat harvest; different uppercase letters (A, B) denote significant differences (**P < 0.01, ***P < 0.001, Tukey's HSD test). Data are means over 2 years. Irrigation applied only in wheat, sorghum was grown as rainfed; CWR: per cent water requirement for wheat; SE_m_ ± : standard error.

**Figure 2:**
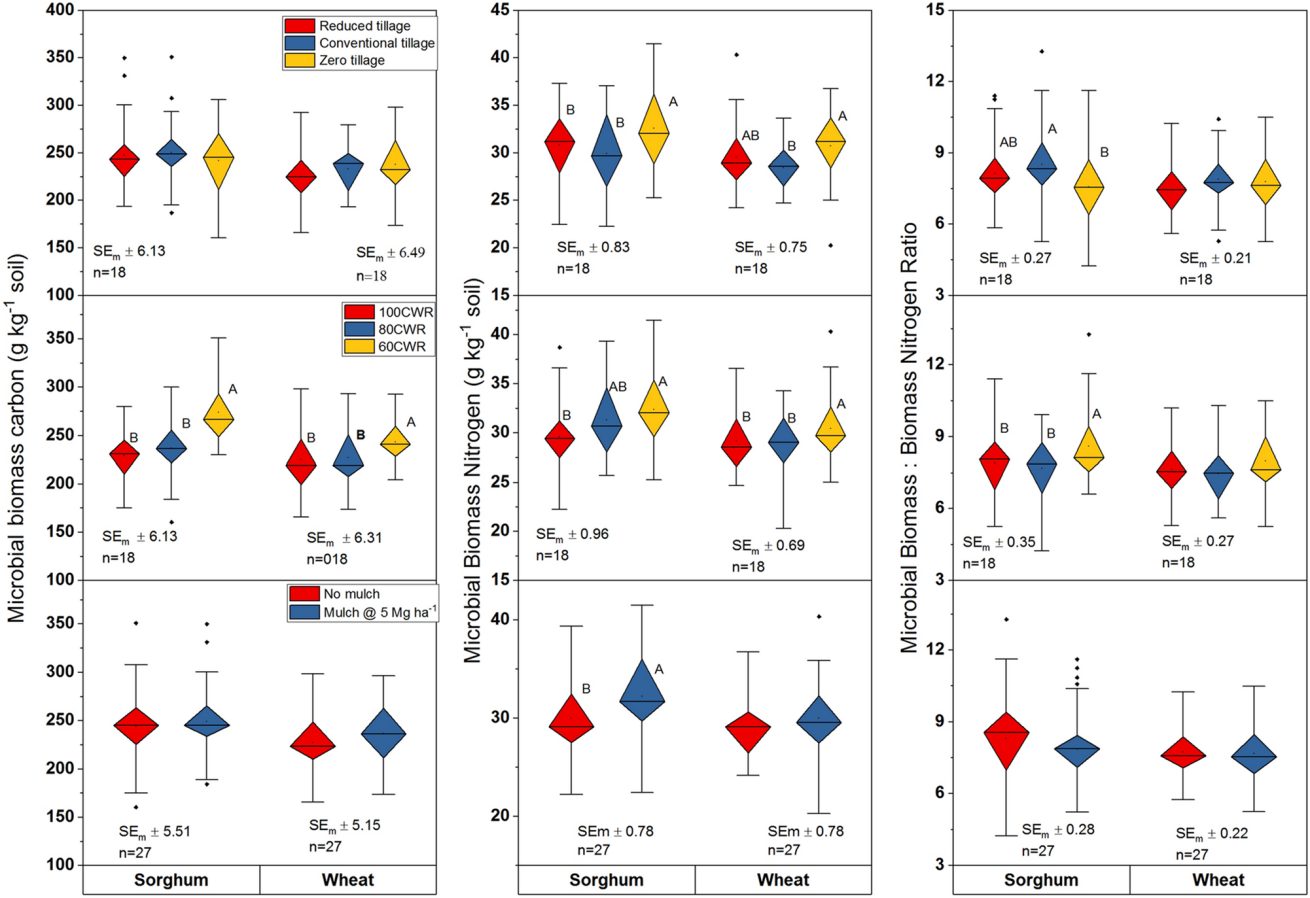
Influence of tillage, irrigation and mulch on dehydrogenase, alkaline phosphatase and urease activities; different uppercase letters (A, B) denote significant differences (**P < 0.01, ***P < 0.001, Tukey's HSD test). Data are means over 2 years. Irrigation applied only in wheat, sorghum was grown as rainfed; CWR: per cent water requirement for wheat; SE_m_ ± : standard error.

The original Article has been corrected.

