# Supplementary material for: Deficit saline water irrigation under reduced tillage and residue mulch improves soil health in sorghum-wheat cropping system in semi-arid region
Source: Sci Rep. 2021 Jan 21;11:1880. doi: 10.1038/s41598-020-80364-4 (PMC7820430; doi:10.1038/s41598-020-80364-4)
Supplement: Supplementary file 1 — Supplementary Information. [file 41598_2020_80364_MOESM1_ESM.doc]

**Supplementary document of “Deficit Saline Water Irrigation under Reduced Tillage and Residue Mulch Improves Soil Health in Sorghum-wheat Cropping System in Semi-arid region”**

**Authors’ information**

Authorline: Pooja Gupta Soni^1,2^, Nirmalendu Basak^1^* Arvind Kumar Rai^1^* , Parul Sundha^1^, Bhaskar Narjary^1^, Parveen Kumar^1^ , Gajender Yadav^1^, Satyendra Kumar^1^ and Rajender Kumar Yadav^1^

^1^ICAR-Central Soil Salinity Research Institute, Karnal 132 001, Haryana, India;

^2^IARI-Krishi Vigyan Kendra, Shikohpur, Gurugram, Pin Code 122 004, Gurugram, Haryana, India

*Corresponding authors email: [nirmalendubasak@rediffmail.com](mailto:nirmalendubasak@rediffmail.com) (N Basak); [AK.Rai@icar.gov.in](mailto:AK.Rai@icar.gov.in) (AK Rai); Telephone and fax no.: +91-0184-2209-401; +91-0184-2290-480

**Supplementary Table 1.** Changes in pH_s_, EC_e_ (dS m^-1^), Walkley Black oxidizable organic C (WBOC; g kg^-1^) and KMnO_4_ oxidizable N, Olsen’s P and NH_4_OAc extractable K (kg ha^-1^), soil microbial biomass carbon and nitrogen (MBC and MBN; g kg^-1^), microbial biomass carbon nitrogen ratio (MBCN), dehydrogenase (DHA, g TPF kg^-1^ day^-1^ soil), alkaline phosphatase (AlK, g *p*-nitrophenol kg^-1^ soil h^-1^), Urease (Ur, g kg^-1^ soil h^-1^), *α*- and *β*-glucosidase (*α*-/*β* glu, g *p*-nitrophenol kg^-1^ soil h^-1^) and on green fodder yield (GFY) and dry fodder sorghum yield (DFSY) (Mg ha^-1^) and weighted linear soil health index (SHI_RS_) in consecutive years

| Soil attributes | pH_s_ | EC_e_ | WBOC | N | P | K | MBC | MBN | MBCN | Dehydrogenase | Alkaline phosphatase | Urease | *α*-glucosidase | *β*-glucosidase |  | | |
| --- | --- | --- | --- | --- | --- | --- | --- | --- | --- | --- | --- | --- | --- | --- | --- | --- | --- |
| Sorghum | | | | | | | | | | | | | | | SHI_RS_ | GFY | DFSY |
| 2015-16 | 7.97 | 5.48^A^ | 3.90 | 89.0 | 23.5 | 247.7 | 246.7 | 29.8^B^ | 7.75^B^ | 89.1^B^ | 23.7 | 3.41^B^ | 3.41^B^ | 14.5 | 0.48^B^ | 55.5 | 13.5 |
| 2016-17 | 7.89 | 3.59^B^ | 3.98 | 86.2 | 23.6 | 243.4 | 246.7 | 32.5^A***^ | 8.40^A^ | 91.8^A^ | 23.6 | 4.39^A***^ | 4.39^A***^ | 14.9 | 0.54^A^ | 55.1 | 13.7 |
| SE_m_± | 0.05 | 0.23 | 0.16 | 1.73 | 0.67 | 3.47 | 5.00 | 0.68 | 0.22 | 0.90 | 0.78 | 0.11 | 0.11 | 0.95 | 0.01 | 3.75 | 0.99 |
| Wheat | | | | | | | | | | | | | | | SHI_IW_ | Grain | Straw |
| 2015-16 | 7.80 | 8.87^A^ | 4.49 | 92.7 | 18.4^B^ | 234.1^A^ | 235.8 | 28.7^B^ | 7.38^B^ | 84.2 | 23.0 | 57.6^B^ | 4.41 | 16.7 | 0.39^B^ | 5.78^B^ | 8.62 |
| 2016-17 | 7.75 | 6.41^B^ | 4.09 | 90.2 | 24.1^A***^ | 213.4^B**^ | 228.3 | 30.5^A**^ | 8.03^A**^ | 84.4 | 23.9 | 59.2^A^ | 4.22 | 16.0 | 0.42^A^ | 6.04^A^ | 8.51 |
| SE_m_± | 0.03 | 0.29 | 0.21 | 1.51 | 0.87 | 4.65 | 5.30 | 0.62 | 0.17 | 1.89 | 0.67 | 0.56 | 0.20 | 1.05 | 0.001 | 0.10 | 0.33 |

Different uppercase letters (A, B) after numeric denote significant differences (** *P*< 0.01, *** *P*< 0.001, Tukey's HSD test); SE_m_±: standard error

**Supplementary Table 2:** Influence of tillage, irrigation and mulch on *α/β*-glucosidasess activities of soils after sorghum and wheat harvest

| Treatments | | *α*-glucosidase | *β*-glucosidase | *α*-glucosidase | | *β*-glucosidase | |
| --- | --- | --- | --- | --- | --- | --- | --- |
|  | | Sorghum | | | Wheat | |  |
| Tillage | Conventional | 3.73 | 13.9 | 4.24 | | 16.4 | |
|  | Reduced | 3.98 | 14.6 | 4.47 | | 16.4 | |
|  | Zero | 3.99 | 15.6 | 4.23 | | 16.2 | |
|  | SE_m_± | 0.13 | 1.16 | 0.25 | | 1.28 | |
| Saline irrigation | 100WR | 3.83 | 14.2 | 4.26 | | 15.8 | |
|  | 80WR | 3.92 | 14.7 | 4.34 | | 16.1 | |
|  | 60WR | 3.94 | 15.2 | 4.35 | | 17.1 | |
|  | SE_m_± | 0.14 | 0.67 | 0.19 | | 0.63 | |
| Mulch | No Mulch | 3.58^B^ | 14.0^B^ | 4.25 | | 16.2 | |
|  | Mulch | 4.22^A***^ | 15.3^A^ | 4.38 | | 16.5 | |
|  | SE_m_± | 0.12 | 0.55 | 0.16 | | 0.51 | |

*α*-/*β*-glucosidase (g *p*-nitrophenol kg^-1^ soil h^-1^); different uppercase letters (A, B) denote significant differences (** *P*< 0.01, *** *P*< 0.001, Tukey's HSD test). Data are means over 2 years. WR: Percent water requirement for wheat, irrigation applied only in wheat, sorghum was grown as rainfed; SE_m_±: standard error

**Supplementary Table 3:** Relationships among the soil parameters after harvest of sorghum under different tillage, mulching and saline irrigation practices

| Soil Properties | GFY | DFY | EC_e_ | pH_s_ | KMnO_4_-N | Olsen’s P | NH_4_OAc K | WBOC | MBC | MBN | MBCN | DHA | *β*-glu | *α*-glu | AlP | Ur |
| --- | --- | --- | --- | --- | --- | --- | --- | --- | --- | --- | --- | --- | --- | --- | --- | --- |
| GFY | 1.00 |  |  |  |  |  |  |  |  |  |  |  |  |  |  |  |
| DFY | 0.84*** | 1.00 |  |  |  |  |  |  |  |  |  |  |  |  |  |  |
| EC_e_ | -0.33 | -0.383 | 1.00 |  |  |  |  |  |  |  |  |  |  |  |  |  |
| pH_s_ | -0.33 | -0.40 | 0.20 | 1.00 |  |  |  |  |  |  |  |  |  |  |  |  |
| KMnO_4_- | 0.29 | 0.33 | 0.09 | -0.11 | 1.00 |  |  |  |  |  |  |  |  |  |  |  |
| Olsen’s P | -0.03 | 0.08 | 0.13 | 0.28 | 0.50* | 1.00 |  |  |  |  |  |  |  |  |  |  |
| NH_4_OAc K | 0.21 | 0.27 | -0.37 | -0.31 | -0.12 | -0.47* | 1.00 |  |  |  |  |  |  |  |  |  |
| WBOC | 0.09 | 0.17 | -0.10 | -0.29 | -0.02 | -0.26 | 0.21 | 1.00 |  |  |  |  |  |  |  |  |
| MBC | 0.46 | 0.35 | -0.25 | -0.32 | -0.18 | -0.32 | 0.43 | 0.24 | 1.00 |  |  |  |  |  |  |  |
| MBN | -0.03 | 0.16 | -0.54** | -0.11 | -0.11 | -0.25 | 0.63* | 0.48* | 0.23 | 1.00 |  |  |  |  |  |  |
| MBCN | 0.42 | 0.16 | 0.16 | -0.16 | -0.11 | -0.14 | -0.076 | -0.14 | 0.71*** | 0.52** | 1.00 |  |  |  |  |  |
| DHA | 0.23 | 0.18 | -0.54** | -0.13 | -0.07 | -0.34 | 0.50* | 0.10 | 0.61** | 0.51** | 0.17 | 1.00 |  |  |  |  |
| *β*-glu | 0.29 | 0.43 | -0.56** | -0.04 | 0.19 | 0.04 | 0.55** | 0.44 | 0.11 | 0.70** | -0.41 | 0.29 | 1.00 |  |  |  |
| *α*-glu | 0.27 | 0.51* | -0.10 | -0.15 | 0.23 | -0.09 | 0.13 | 0.61** | 0.23 | 0.29 | -0.01 | 0.14 | 0.50* | 1.00 |  |  |
| AlP | 0.07 | -0.17 | 0.18 | -0.23 | -0.41 | -0.64** | 0.04 | -0.01 | 0.44 | -0.25 | 0.59** | 0.26 | -0.51** | 0.01 | 1.00 |  |
| Ur | 0.21 | 0.005 | 0.30 | -0.06 | 0.14 | -0.14 | -0.184 | 0.45* | -0.17 | -0.007 | -0.12 | -0.42 | 0.11 | 0.21 | 0.07 | 1.00 |

[*Correlation is significant at the 0.05 level (2-tailed); **Correlation is significant at the 0.01 level (2-tailed);***Correlation is significant at the 0.001 level (2-tailed); GFY: green fodder yield; DFSY: dry fodder sorghum yield; EC_e_; electrical conductivity of soil water saturation paste extract; pH_s_: pH soil water saturation; MBC: microbial biomass C; MBN: microbial biomass N; microbial biomass carbon nitrogen ratio (MBCN); DHA: dehydrogenase activity; *β*-glu: *β*-glucosidase activity; *α*-glu: *α*-glucosidase activity; AlP: Alkaline phosphatase activity; Ur: Urease activity]

**Supplementary Table 4:** Relationships among the soil parameters after harvest of wheat under different tillage, mulching and saline irrigation practices

| Soil Properties | Wheat grain | Wheat straw | EC_e_ | pH_s_ | KMnO_4_-N | Olsen’s P | NH_4_OAc K | WBOC | MBC | MBN | MBCN | DHA | *β*-glu | *α*-glu | AlP | Ur |
| --- | --- | --- | --- | --- | --- | --- | --- | --- | --- | --- | --- | --- | --- | --- | --- | --- |
| Wheat grain | 1.00 |  |  |  |  |  |  |  |  |  |  |  |  |  |  |  |
| Wheat straw | 0.72*** | 1.00 |  |  |  |  |  |  |  |  |  |  |  |  |  |  |
| EC_e_ | -0.36 | -0.18 | 1.00 |  |  |  |  |  |  |  |  |  |  |  |  |  |
| pHs | 0.21 | 0.12 | 0.052 | 1.00 |  |  |  |  |  |  |  |  |  |  |  |  |
| KMnO_4_-N | 0.212 | -0.07 | -0.157 | 0.077 | 1.00 |  |  |  |  |  |  |  |  |  |  |  |
| Olsen’s P | 0.147 | -0.177 | 0.057 | -0.177 | 0.017 | 1.00 |  |  |  |  |  |  |  |  |  |  |
| NH_4_OAc K | -0.04 | -0.404 | 0.18 | -0.308 | 0.048 | 0.36 | 1.00 |  |  |  |  |  |  |  |  |  |
| WBOC | -0.12 | -0.30 | -0.50* | -0.32 | 0.49* | 0.01 | 0.28 | 1.00 |  |  |  |  |  |  |  |  |
| MBC | 0.12 | -0.322 | -0.212 | 0.26 | -0.16 | 0.166 | 0.42 | 0.04 | 1.00 |  |  |  |  |  |  |  |
| MBN | -0.23 | -0.33 | -0.02 | -0.19 | -0.03 | 0.16 | 0.47* | 0.37 | 0.44 | 1.00 |  |  |  |  |  |  |
| MBCN | 0.09 | -0.22 | -0.01 | 0.25 | -0.31 | 0.12 | 0.109 | -0.22 | 0.72*** | 0.06 | 1.00 |  |  |  |  |  |
| DHA | 0.47* | 0.19 | -0.49* | 0.19 | 0.20 | 0.14 | 0.33 | 0.31 | 0.51* | 0.34 | 0.042 | 1.00 |  |  |  |  |
| *β*-glu | 0.14 | 0.16 | -0.21 | -0.07 | -0.06 | 0.10 | 0.21 | 0.13 | 0.31 | 0.38 | -0.07 | 0.42 | 1.00 |  |  |  |
| *α*-glu | 0.31 | 20 | -0.24 | -0.03 | 0.14 | 0.50* | -0.08 | 0.28 | -0.22 | -0.05 | -0.26 | 0.16 | -0.13 | 1.00 |  |  |
| AlP | -0.30 | -0.001 | 0.13 | -0.18 | -0.45 | 0.10 | -0.41 | -0.26 | -0.06 | 0.05 | 0.24 | -0.12 | -0.08 | -0.11 | 1.00 |  |
| Ur | 0.46 | 0.22 | -0.56* | 0.15 | 0.31 | 0.37 | 0.09 | 0.22 | 0.03 | -0.13 | -0.17 | 0.32 | 0.08 | 0.37 | -0.52* | 1.00 |

[*Correlation is significant at the 0.05 level (2-tailed); **Correlation is significant at the 0.01 level (2-tailed);***Correlation is significant at the 0.001 level (2-tailed). GFY: green fodder yield; DFY: dry fodder yield; EC_e_; electrical conductivity of soil water saturation paste extract; pH_s_: pH soil water saturation; MBC: microbial biomass C; MBN: microbial biomass N; MBCN: microbial biomass carbon nitrogen ratio; DHA: dehydrogenase activity; *β*-glu: *β*-glucosidase activity; *α*-glu: *α*-glucosidase activity; AlP: Alkaline phosphatase activity; Ur: Urease activity]

**Supplementary Table 5:** Principal component analysis of soil attributes after sorghum harvest

| Statistics | PC1 | PC2 | PC3 | PC4 | PC5 | PC6 |
| --- | --- | --- | --- | --- | --- | --- |
| Eigenvalue | 2.38 | 1.96 | 1.61 | 1.28 | 1.20 | 0.99 |
| Per cent | 17.01 | 13.97 | 11.51 | 9.14 | 8.58 | 7.08 |
| Cum Percent | 17.01 | 30.98 | 42.49 | 51.64 | 60.22 | 67.30 |
| Eigen vectors |  |  |  |  |  |  |
| MBCN: microbial biomass carbon nitrogen ratio | 0.34 | 0.32 | -0.40 | **0.55** | **0.55** | **0.53** |
| *α*-glucosidase activity | **0.41** | -0.04 | 0.29 | -0.15 | -0.27 | 0.41 |
| pH_s_ | -0.16 | -0.29 | 0.22 | -0.11 | -0.06 | 0.32 |
| NH_4_OAc K | 0.01 | 0.02 | -0.38 | 0.39 | 0.32 | 0.23 |
| EC_e_ | -0.36 | **0.44** | -0.22 | 0.32 | 0.28 | 0.20 |
| KMnO_4_-N | -0.12 | **-0.45** | 0.20 | -0.06 | 0.04 | 0.12 |
| Dehydrogenase activity | 0.36 | 0.30 | 0.28 | -0.14 | -0.08 | 0.11 |
| Microbial biomass C | **0.39** | 0.05 | 0.15 | -0.04 | 0.15 | 0.10 |
| *β*-glucosidase  activity | 0.27 | -0.09 | 0.05 | 0.08 | 0.23 | 0.09 |
| Microbial biomass N | 0.31 | 0.33 | **0.43** | -0.44 | -0.35 | -0.07 |
| Olsen’s P | -0.13 | -0.32 | -0.05 | 0.17 | 0.25 | -0.15 |
| Alkaline phosphatase activity | 0.03 | -0.02 | -0.17 | 0.23 | 0.26 | -0.17 |
| WBOC | 0.28 | -0.06 | 0.12 | 0.04 | 0.17 | -0.34 |
| Urease activity | -0.04 | 0.32 | 0.38 | -0.32 | -0.28 | -0.37 |

EC_e_; electrical conductivity of soil water saturation paste extract; pH_s_: pH soil water saturation; Bold values indicate highly weighted variables for the respective principal components (PC)

**Supplementary Table 6:** Principal component analysis of soil attributes after wheat harvest

| Statistics | PC1 | PC2 | PC3 | PC4 | PC5 | PC6 |
| --- | --- | --- | --- | --- | --- | --- |
| Eigenvalue | 2.29 | 1.78 | 1.27 | 1.21 | 1.08 | 1.01 |
| Per cent | 16.34 | 12.72 | 9.10 | 8.66 | 7.74 | 7.19 |
| Cum Per cent | 16.34 | 29.05 | 38.15 | 46.81 | 54.55 | 61.74 |
| Eigen vectors |  |  |  |  |  |  |
| EC_e_ | **-0.42** | 0.24 | 0.17 | -0.37 | 0.14 | **0.62** |
| WBOC | -0.27 | 0.21 | -0.18 | -0.03 | -0.11 | 0.37 |
| Dehydrogenase activity | 0.18 | 0.33 | -0.11 | 0.37 | 0.22 | 0.25 |
| *β*-glucosidase  activity | -0.04 | 0.289 | -0.18 | 0.26 | -0.19 | 0.21 |
| *α*-glucosidase activity | -0.20 | 0.16 | -0.37 | -0.36 | -0.05 | 0.18 |
| NH_4_OAc K | -0.21 | 0.38 | 0.22 | 0.003 | 0.03 | 0.15 |
| Microbial biomass C | 0.26 | **0.54** | -0.12 | -0.20 | 0.19 | 0.14 |
| pH_s_ | -0.09 | -0.05 | 0.16 | 0.22 | 0.23 | 0.12 |
| Microbial biomass N | 0.36 | 0.18 | -0.16 | 0.13 | 0.15 | 0.10 |
| Urease activity | -0.06 | 0.19 | **0.56** | -0.17 | -0.56 | -0.06 |
| Alkaline phosphatase activity | 0.26 | -0.17 | 0.18 | -0.17 | -0.20 | -0.11 |
| Olsen’sP | 0.405 | -0.10 | -0.32 | 0.35 | 0.22 | -0.25 |
| MBCN: microbial biomass carbon nitrogen ratio | **0.42** | 0.31 | 0.08 | -0.24 | **0.56** | -0.30 |
| KMnO_4_-N  N | -0.13 | 0.21 | 0.45 | **0.43** | -0.24 | -0.32 |

EC_e_; electrical conductivity of soil water saturation paste extract; pH_s_: pH soil water saturation;

Bold values indicate highly weighted variables for the respective principal components (PC)

**Table 7.** Initial soil physicochemical properties of the experimental field

| **Soil properties** | **Soil depth 0-15 cm** |
| --- | --- |
| Texture | Sandy loam |
| pH_s_ | 8.2 ± 0.2 |
| EC_e_ (dS m^-1^) | 16.2 ± 9.6 |
| Organic carbon (%) | 0.46 ± 0.1 |
| KMnO_4_-N kg ha^-1^ | 132.0 ± 7.9 |
| Olsen’s P kg ha^-1^ | 40.1 ± 3.5 |
| NH_4_OAcK kg ha^-1^ | 292.3 ± 10.6 |
| Cation exchange capacity cmol_(P+)_ kg^-1^ | 13.4 ± 3.25 |
| Exchangeable sodium per cent (ESP) % | 39.3 ± 12.47 |
| Micronutrients (mg kg^-1^): DTPA extractable |  |
| Fe | 9.6 ± 0.6 |
| Mn | 1.9 ± 0.08 |
| Cu | 0.57 ± 0.02 |
| Zn | 1.22 ± 0.15 |

[pH_s_: pH soil water saturation; EC_e_: electrical conductivity of soil water saturation paste extract]

**Table 8:** Composition of irrigation water

| Parameter |  | Groundwater |
| --- | --- | --- |
| EC_iw_ (dS m^-1^) |  | 7.99 |
| pH_iw_ |  | 7.4 |
| Ca^2+^ + Mg^2+^ (me L^-1^) |  | 31.5 |
| Na^+^ (me L^-1^) |  | 74.2 |
| K^+^ (me L^-1^) |  | 1.5 |
| CO_3_^-^ (me L^-1^) |  | ND |
| HCO_3_^-^ (me L^-1^) |  | 5.4 |
| Cl^-^ (me L^-1^) |  | 48.9 |
| SO_4_^2-^ (me L^-1^) |  | 52.8 |
| SAR ( mmol^1^*/*^2^ L^−1^*/*^2^ ) |  | 18.7 |

[EC_iw_, electrical conductivity of irrigation water; pH_iw_: pH of irrigation water; ND; Non detectable; SAR: Na^+^/[(Ca^2+^ + Mg^2+^)/2]^1/2^ sodium adsorption ratio in mmol^1^*/*^2^ L^−1^*/*^2^]


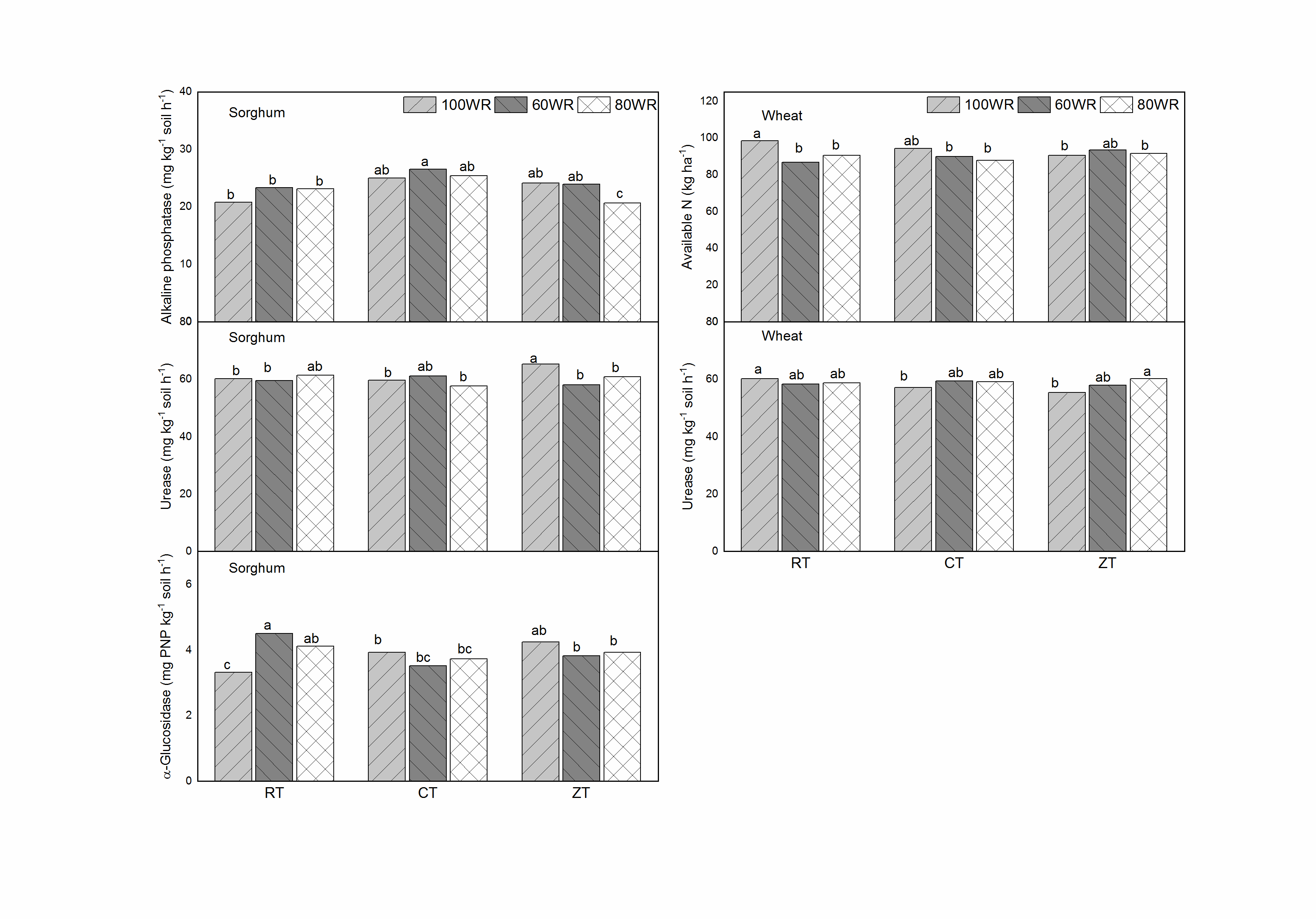


Supplementary Fig.1. Interaction effect of tillage × saline irrigation on alkaline phosphatase, urease (Ur) and *α*- glucosidase activities after sorghumt; available N and urease activitiy after wheat; the bars with same letters are not statistically different (*P*≤ 0.05, Tukey's HSD test).


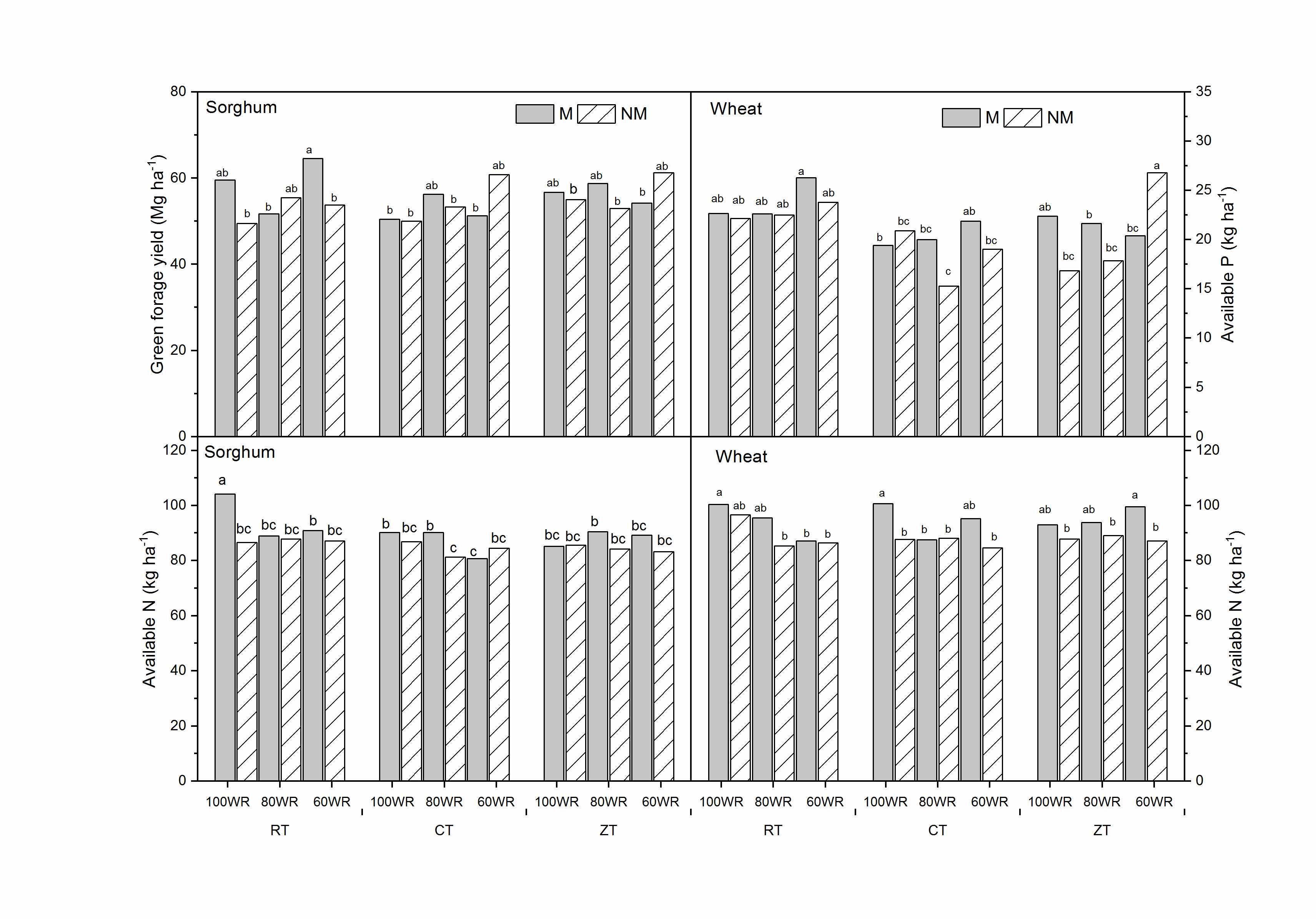


Supplementary Fig.2. Interaction effect of tillage × saline irrigation × mulch on green fodder yield of sorghum; available P after wheat; and available N after sorghum and wheat; the bars with same letters are not statistically different (*P*≤ 0.05, Tukey's HSD test).


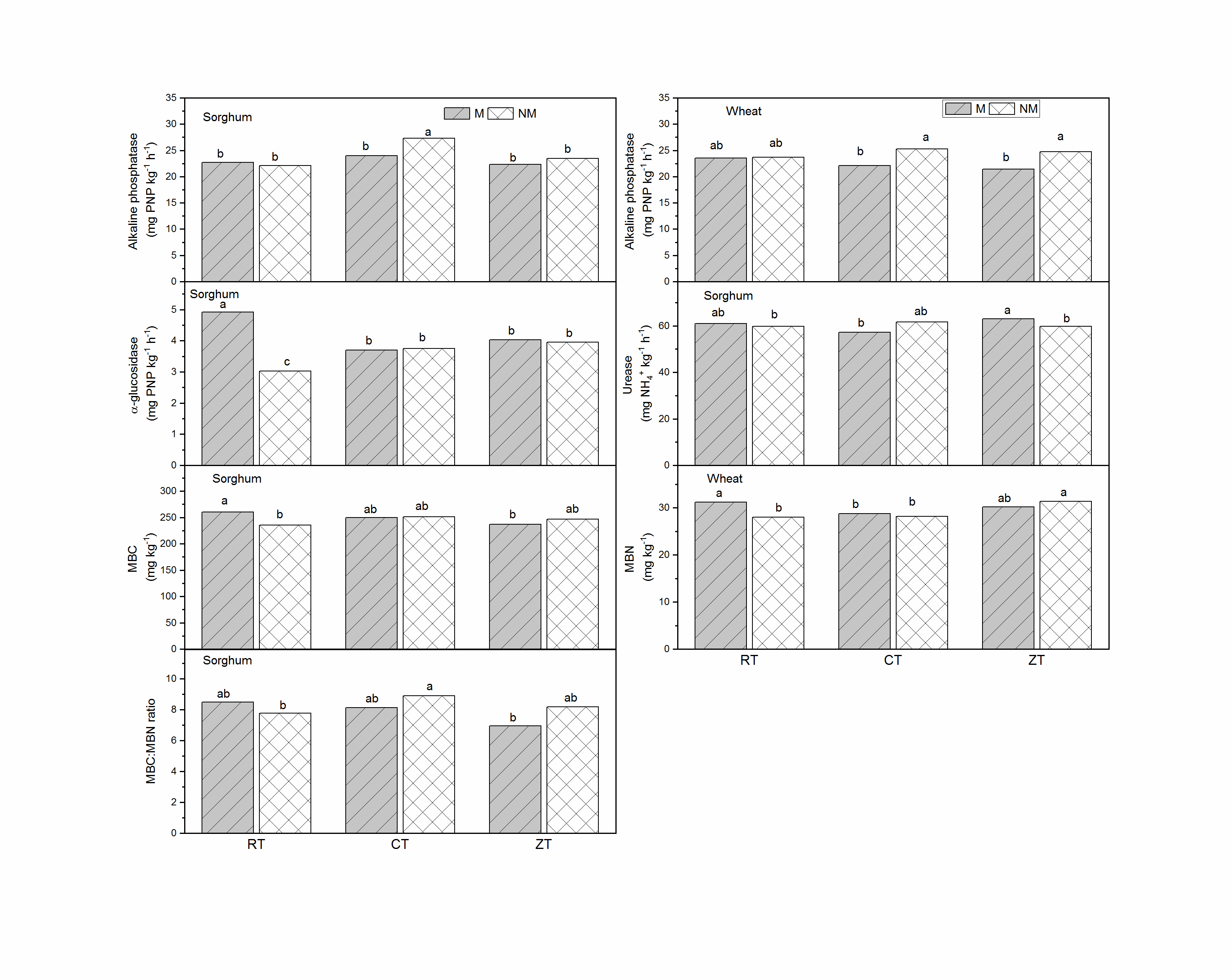


Supplementary Fig.3. Interaction effect of tillage × mulch on alkaline phosphatase, *α*- glucosidase activity, microbial biomass C (MBC), MBC; MBN ratio (MBCN) and urease (Ur) activity after sorghum harvest; microbial biomass N (MBN) and alkaline phosphatase activity after wheat; the bars with same letters are not statistically different (*P*≤ 0.05, Tukey's HSD test).


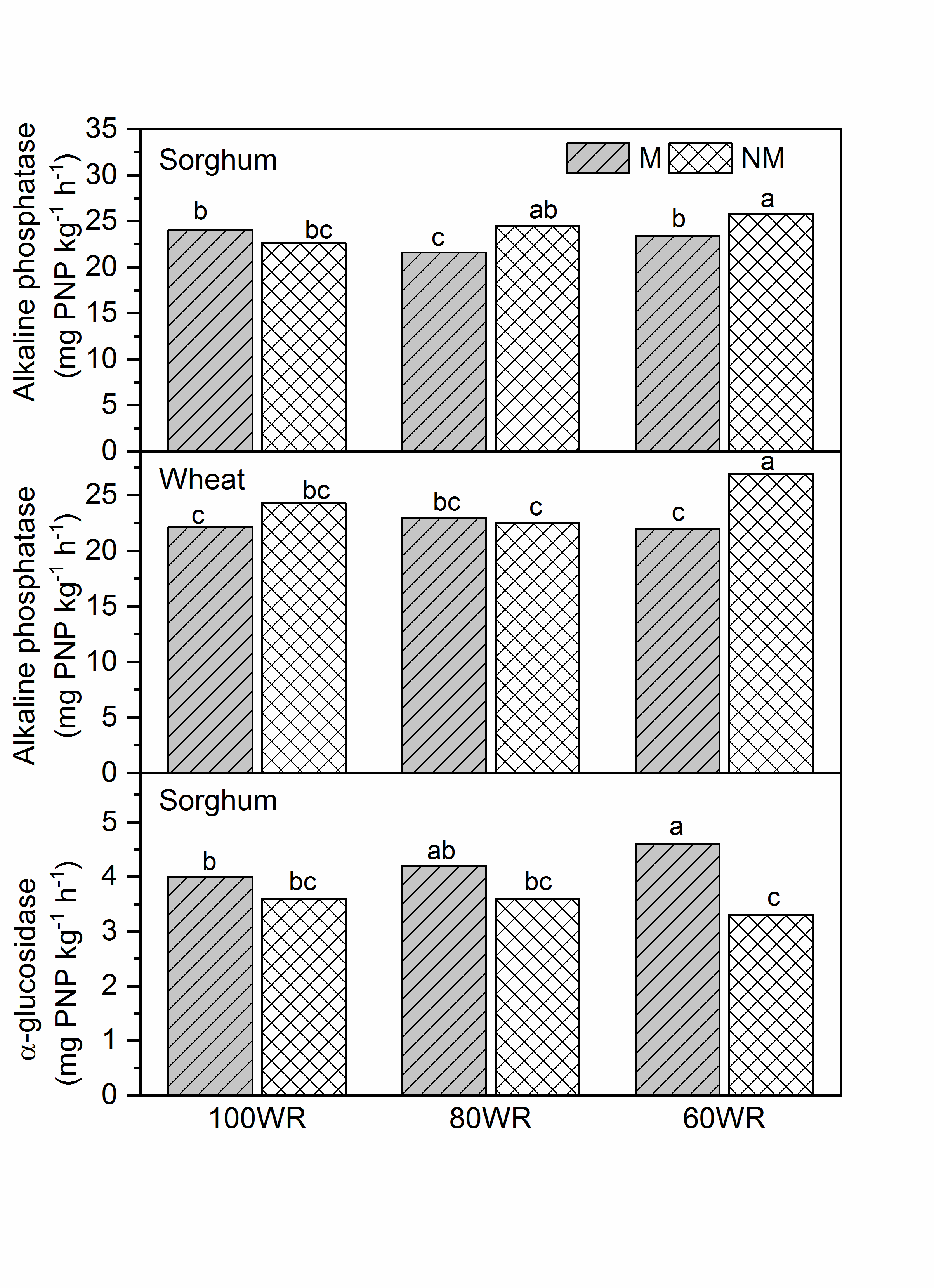


Supplementary Fig.4. Interaction effect of irrigation × mulch on alkaline phosphatase and *α*- glucosidase activities after sorghum; alkaline phosphatase after wheat; the bars with same letters are not statistically different (*P*≤ 0.05, Tukey's HSD test).


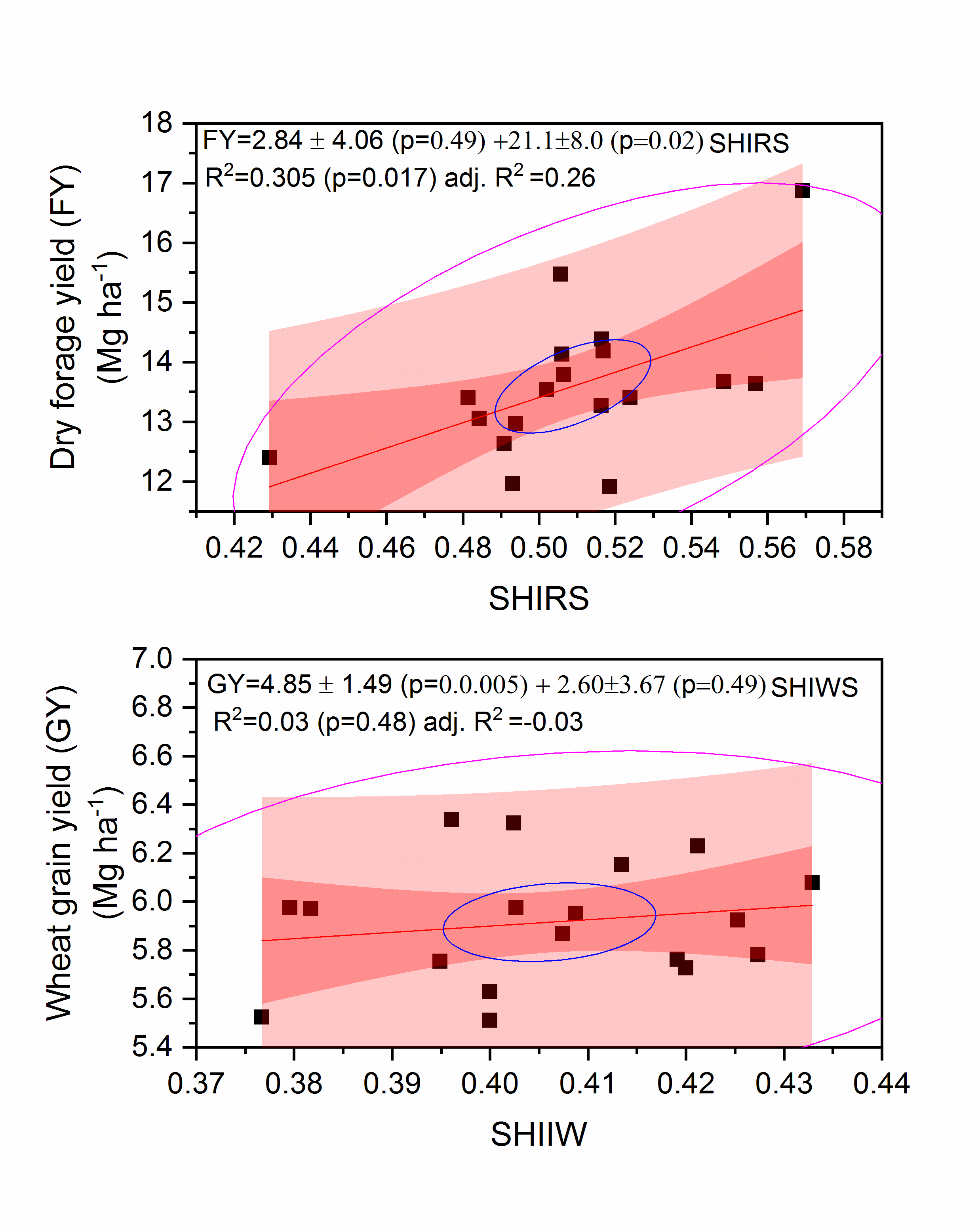


Supplementary Fig.5. Relationship betweens SHI_RS_ (Soil health index for rainfed sorghum) and sorghum dry fodder yield SHI_IW_ (Soil health index for irrigated wheat) and wheat grain yield.


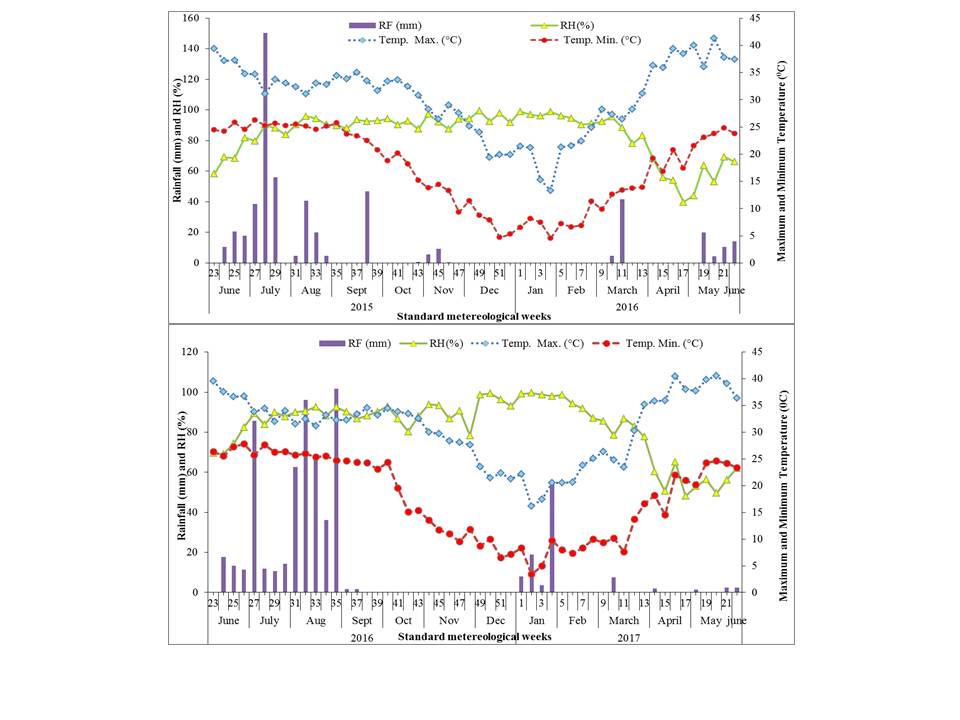


Supplementary Fig.6. Average weekly weather data during 2015-16 and 2016-17 (RF-rainfall; RH- relative humidity; Temp. Max.- maximum temperature; Temp. Min.- minimum temperature).
